# Functional Interfaces, Biological Pathways, and Regulations of Interferon-Related DNA Damage Resistance Signature (IRDS) Genes

**DOI:** 10.3390/biom11050622

**Published:** 2021-04-22

**Authors:** Monikaben Padariya, Alicja Sznarkowska, Sachin Kote, Maria Gómez-Herranz, Sara Mikac, Magdalena Pilch, Javier Alfaro, Robin Fahraeus, Ted Hupp, Umesh Kalathiya

**Affiliations:** 1 International Centre for Cancer Vaccine Science, University of Gdansk, ul. Kładki 24, 80-822 Gdansk, Poland; alicja.sznarkowska@ug.edu.pl (A.S.); sachin.kote@ug.edu.pl (S.K.); herranzmg@gmail.com (M.G.-H.); sara.mikac@phdstud.ug.edu.pl (S.M.); s1797808@sms.ed.ac.uk (M.P.); javier.alfaro@proteogenomics.ca (J.A.); robin.fahraeus@inserm.fr (R.F.); ted.hupp@ed.ac.uk (T.H.); 2 Institute of Genetics and Molecular Medicine, University of Edinburgh, Edinburgh EH4 2XR, UK; 3 Inserm UMRS1131, Institut de Génétique Moléculaire, Université Paris 7, Hôpital St. Louis, F-75010 Paris, France; 4 Department of Medical Biosciences, Building 6M, Umeå University, 901 85 Umeå, Sweden; 5 RECAMO, Masaryk Memorial Cancer Institute, Zlutykopec 7, 65653 Brno, Czech Republic

**Keywords:** DNA damage, IRDS genes, DNA, RNA, ATP, functional site, viruses, receptors, resistance, interferon, chemotherapy and radiotherapy, protein interfaces, upstream regulator

## Abstract

Interferon (IFN)-related DNA damage resistant signature (IRDS) genes are a subgroup of interferon-stimulated genes (ISGs) found upregulated in different cancer types, which promotes resistance to DNA damaging chemotherapy and radiotherapy. Along with briefly discussing IFNs and signalling in this review, we highlighted how different IRDS genes are affected by viruses. On the contrary, different strategies adopted to suppress a set of IRDS genes (STAT1, IRF7, OAS family, and BST2) to induce (chemo- and radiotherapy) sensitivity were deliberated. Significant biological pathways that comprise these genes were classified, along with their frequently associated genes (IFIT1/3, IFITM1, IRF7, ISG15, MX1/2 and OAS1/3/L). Major upstream regulators from the IRDS genes were identified, and different IFN types regulating these genes were outlined. Functional interfaces of IRDS proteins with DNA/RNA/ATP/GTP/NADP biomolecules featured a well-defined pharmacophore model for STAT1/IRF7-dsDNA and OAS1/OAS3/IFIH1-dsRNA complexes, as well as for the genes binding to GDP or NADP+. The Lys amino acid was found commonly interacting with the ATP phosphate group from OAS1/EIF2AK2/IFIH1 genes. Considering the premise that targeting IRDS genes mediated resistance offers an efficient strategy to resensitize tumour cells and enhances the outcome of anti-cancer treatment, this review can add some novel insights to the field.

## 1. Introduction

**Figure 1:**
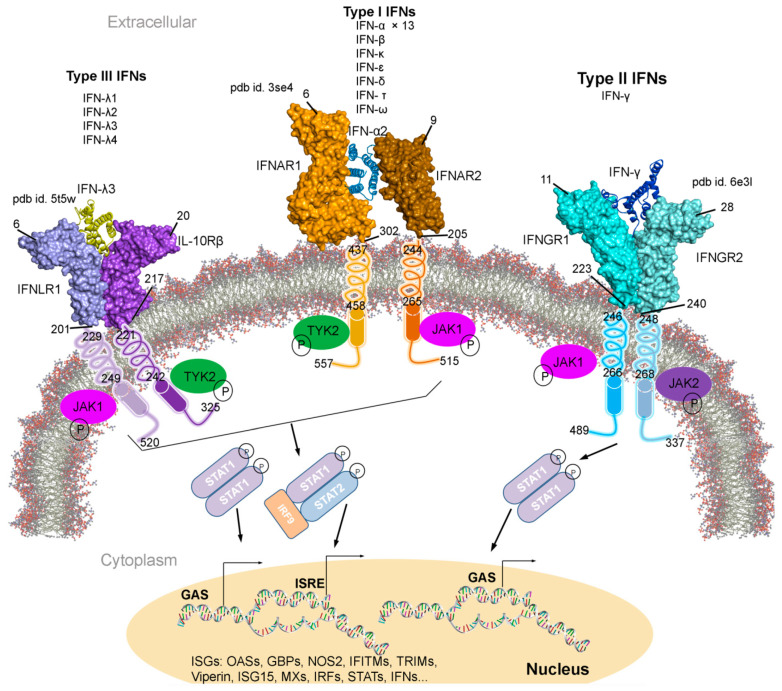
Type I, II, and III interferons (IFNs) signalling cascades, along with their tertiary structures retrieved from the Protein data bank (PDB) [7] database. Individual IFN receptors from the IFNAR1-IFNα2-IFNAR2 (pdb id. 3se4 [17]), IFNGR1-IFNγ-IFNGR2 (pdb id. 6e3l [18]), and IFNLR1-IFNλ3-IL10Rβ (pdb id. 5t5w [19]) complexes containing the extracellular topological domain (ETD), transmembrane domain (TMD) and cytoplasmic domain (CPD) are described with their amino acid range. Type I, II and III IFNs signal via distinct receptors IFNAR (composed of IFNAR1 and IFNAR2), IFNGR (IFNGR1 and IFNGR2) and IFNLR (INFLR1 and IL-10Rβ), respectively [20,21]. Upon the IFN binding to the receptor complex, JAK1 (Janus kinase 1) and TYK2 (tyrosine kinase 2) are activated by cross-phosphorylation within the cytoplasmic regions of the receptor, which then phosphorylate STAT1 and STAT2 (signal transducer and activator of transcription 1 and 2). STATs from various complexes migrate to the nucleus and binds the IFN-stimulated response elements (ISREs) or gamma-activated sequences (GASs), which leads to the activation of transcription of several genes involved in antiviral responses comprising ISGs, IFNs, IRFs and STATs [20,21]. Abbreviations: IFNAR, interferon alpha and beta receptor; IFNGR, interferon-gamma receptor; IFNLR, interferon lambda receptor; L10Rβ, interleukin 10 receptor Beta; aa, amino acids; P, phosphate; OASs, oligoadenylate synthases; GBPs, guanylate binding proteins; NOS2, nitric oxide synthase 2; IFITMs, IFN-induced transmembrane proteins; and TRIMs, tripartite motif proteins. Visualization and representation of the tertiary structures of IFNs and its associated receptors was performed using the BIOVIA Discovery Studio Visualizer (Dassault Systèmes, BIOVIA, San Diego, CA, USA), IFNs are shown in ribbon and its receptors as surface.

**Figure 2:**
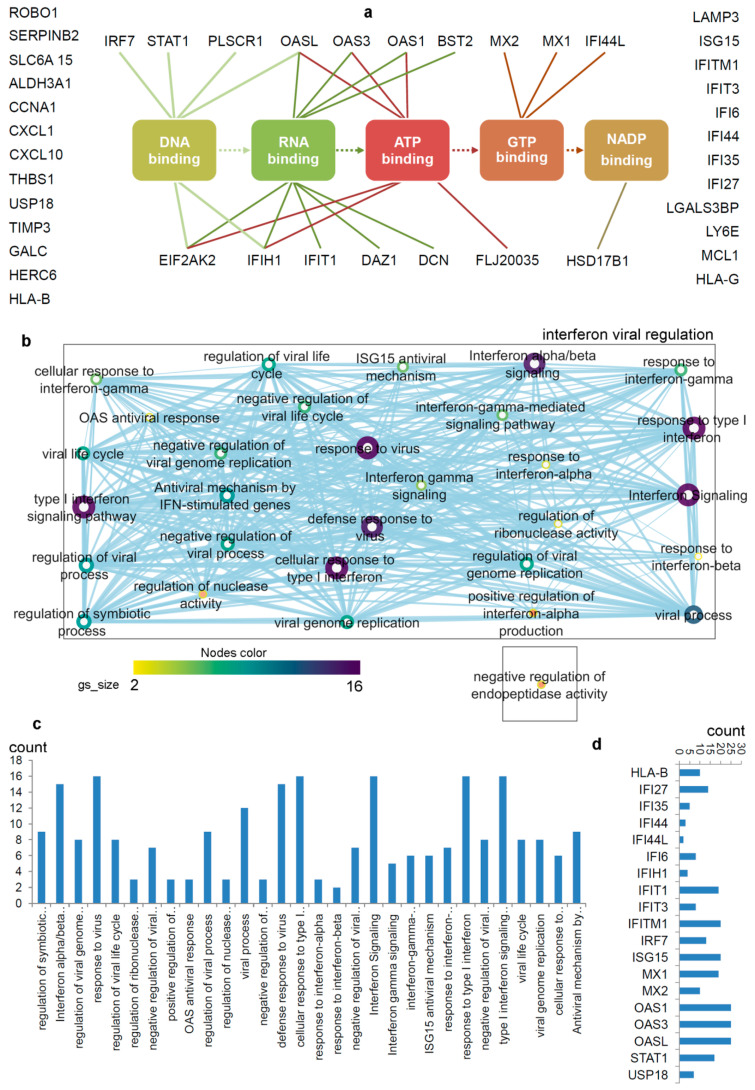
Differentiating IRDS genes considering their binding molecules, and associated significant biological pathways [71,74,75]. (**a**) A network describing IRDS genes involvement in binding with diverse biomolecules; DNA, RNA, ATP, GTP or NADP [77]. (**b**) Pathway enrichment analysis for the IRDS genes listed in the panel (**a**). The enrichment analysis was performed using g:Profiler [78,79], and as the following step, the networks were visualized using the “Enrichment Map” platform in the Cytoscape package [80]. Parameters for the pathway enrichment analysis in g:Profiler and Cytoscape were set as described in the protocol by Reimand et al. [79]. (**c**) The total number of IRDS genes involved in a particular pathway. (**d**) Frequency of IRDS genes occurring in different pathways that are identified in panel (**c**), and only genes with higher frequency are presented. For pathway analysis as shown in panel (**b**), the node size corresponds to the number of genes in the dataset/gene-set size, and colour of the node corresponds to the number of the geneset for the dataset. Edge size corresponds to the number of genes that overlap between two connected genesets. Intra- and interconnecting nodes means some genes are shared in clusters or pathways and, hence, they are represented as edges. In the cytoscape, following parameters were set for the plots: the chart data, Q-value (FDR) columns; and chart type, radial heat map (RdBu-3). The plots and networks between pathways for this figure were generated with the data retrieved from the Cytoscape program [80].

Radiotherapy is an effective treatment for many cancer types, which is used in ~60% cancer patients and frequently with surgery or chemotherapy [1]. A decade of efforts has demonstrated that the Type 1 interferon (IFN-I; Figure 1) cytokine system is compelling in mediating the efficacy of radiotherapy [2,3]. Type 1 IFNs produced by irradiated tumour cells and tumour-infiltrating leukocytes can enhance dendritic cell cross-priming of CD8+ T-cells (cytolytic T cell) and concomitant T-cell mediated killing. These IFN-I may act upon irradiated cancer cells directly, priming them for immunogenic, and necroptotic cell death [4,5]. The mechanisms by which Type 1 IFNs promote the efficacy of standard-of-care cancer treatments, remains an area of active investigation. Nevertheless, tumours often develop resistance to radiotherapy, and paradoxically, recent work has shown that elevated tumoural expression of a subset of IFN-stimulated genes (ISGs) known as the IFN-related DNA damage resistance signature (IRDS) positively correlates with therapy resistance across multiple cancer types [1,6]. This review highlights proteins encoded as the IRDS genes, with assessing their known tertiary structures from the protein data bank (PDB) database [7] and functional interfaces (Figure 2a) with DNA, RNA, ATP, (adenosine triphosphate), GTP (guanosine triphosphate) or NADP+ (nicotinamide adenine dinucleotide phosphate). The regulation of antiviral effects by IFNs and different ways adopted by viruses to evade IFNs induced antiviral responses are discussed, as well as investigating IRDS genes enrichments in different biological pathways. Subsequently, the diverse set of IRDS inhibitory treatments practiced are addressed, with considering the fact that targeting IRDS mediated resistance offers an efficient strategy to resensitize tumour cells.

### 1.1. Interferon Types and Their Signaling Cascade

Upon recognition of viral, pathogenic, and tumorigenesis the activated immune system synthesizes different cytokines or interferons [8]. Along the viral and tumour suppression activities by IFNs, their anti-proliferative, pro-apoptotic and immunomodulatory roles have also been characterized [9,10,11,12]. The interferon family is classified in three different types (Figure 1); IFN-I (IFNα-1/13, IFNβ, IFNε, IFNκ and IFNω), IFN-II (IFNγ), and IFN-III (IFNλ-1/4) [13,14]. IFN-I are homologous cytokines [9], and particularly, the IFNα genes are expressed by most nucleated cell types. Despite different redundant roles, the IFN-I is typically secreted as a result of infection (IFNβ, released by non-immune cells; and IFNα, secreted by immune cells) [10,15], whereas IFN-II is essentially activated upon immune and inflammatory stimuli [16].

Individual interferon bind specifically to a particular IFN receptor type, which results in the activation of the signalling cascade. All subtypes of IFN-I with different affinities binds to the same cell surface receptors (from class II cytokine receptor family); IFNAR1 and IFNAR2 (interferon alpha and beta receptor subunit 1 and 2) [22,23,24,25]. Generated IFNAR1-IFN-IFNAR2 complex, mediates the activation of different signal transduction through the JAK/STAT pathway, which leads to the regulation of several genes (Figure 1) [9,25,26]. Particularly, the IFNAR2 is assumed to be responsible for the affinity determination and differential recognition of Type I IFNs, and IFNAR1 receptor is implicated in a discrete set of signal transduction pathways and upon the ligand binding (Figure 1). Consequently, two members of the Janus tyrosine kinases family, TYK2 (tyrosine kinase 2) and JAK1 (Janus kinase 1), associate to the cytoplasmic domain of the IFNAR1 and IFNAR2, respectively. Sequentially, the STAT is phosphorylated forming STAT1-STAT2 heterodimers in IFN-I, whereas the STAT1-STAT1 homodimer are formed in IFN-II and translocated to the nucleus (Figure 1) [27]. IFN-I and IFN-II activation are interrelated (Figure 1); suppressing IFN-I signal decreases IFN-II effects, similarly enhancing IFN-I increases IFN-II response [28,29,30,31].

Rising number of effector molecules are being characterized, with summarizing their roles in antiviral responses, DNA-replication (p53, c-myc), RNA-turnover (2-5A-synthetase, RNAse L) and protein translation (*M-Tor*, PKR) [9]. Depending on the test system and readouts employed, the potency of the IFNα subtypes varies significantly, however, IFNα8 is often claimed to be the most potent and IFNα1 has the weakest activity [32,33]. In addition, to the potentially regulating antiviral effects, IFNs act directly or indirectly in both innate and adaptive immune responses, on natural killer (NK) cells, T cells, B cells, dendritic cells (DCs) and phagocytic cells [34]. Furthermore, despite condition-dependent behaviour, IFNs are repeatedly used in different treatments, for example, presently available anti-HBV (Hepatitis B virus) and anti-HCV (Hepatitis C virus) treatments are based on the administration of Type I IFNs. Therapies with the recombinant subtypes IFNα- 2a or 2b, and their chemically modified derivatives; PEG-IFNs (pegylated interferon), are current clinical therapeutics standards. For HBV, PEG-IFN2a is recommended as the first choice, and HCV treatment regimens are based on PEG-IFN 2a or 2b combined with the nucleoside analogueribavirin as ‘state of the art’ treatment. 

### 1.2. Downstream Events for the Cellular Response against DNA Damage

In vivo studies proved that chemotherapy and radiation treatment induce Type I IFN signalling in tumours to develop anti-tumour immunity [2,35,36], as well as the mechanistic insights into how DNA damage induces Type I IFNs have been classified [6,37]. The strong crosstalk between immune responses and DNA damage response (DDR) has been found for the recognition of misplaced self-DNAs. The DNA damage can trigger innate immune response through the accumulation of nuclear DNA in the cytoplasm, a common characteristic of tumours and cancer cell lines in the accumulation of cytoplasmic ssDNA or dsDNA [38,39,40]. Many human tumours display chromosomal instability (CIN) phenomenon, often coincides with cytosolic DNA, which activates the cyclic guanosine monophosphate (GMP)-adenosine monophosphate (AMP) synthase (cGAS)-STING pathway signalling, forming the central node between cancer cells and its surrounding microenvironment [40,41].

cGAS-STING is a major cytosolic ssDNA and dsDNA sensor, the receptor protein GMP-AMP synthase sense cytosolic dsDNA and synthesizes secondary messenger 2′,3′-cyclic GMP-AMP. The latter one is detected by the downstream sensor protein STING, triggering IFN regulatory factor 3 (IRF3) activation for the Type I IFN production. In further detail, the cGAS activation is regulated by dsDNA in a length-dependent manner, as long DNA fragments (in range of kilobase) activate cGAS more efficiently compared to shorter ones. Likewise, cGAS also guides the downstream production of IFN with a longer portion of DNA being more immunostimulatory [42]. The cGAS-STING signalling cascade upregulates Type I IFN expression and such response activates the innate immune system, particularly the NK cells, which increases their cytotoxic activity [43]. The binding of the canonical Type I IFNs; IFNα or IFNβ to their receptors IFNAR or IFNBR, respectively, leads to the JAK1 and TYK2 activation, as well as the downstream recruitment, dimerization, and nuclear translocation of STAT proteins [15]. The STAT1 gene binds to INFγ-activated sequences to promote the transcription of pro-inflammatory genes, for instance IRF1 and CXCL9 [44]. On the other hand, STAT3 homodimers formed following the activation of JAK-JAK receptors, act as suppressors of pro-inflammatory genes [45]. Parallel to STAT1 activation, cGAS-STING activity also facilitates STAT3 activity that counteracts STAT1-mediated activation of NK cells in a negative feedback loop [46]. Moreover, STAT3 activity reduces the migration of various immune cells to the tumour microenvironment involving NK cells, T cells, neutrophils, and macrophages, further contributes to an immunosuppressive tumour microenvironment. Contrarily, inhibiting STAT3 activity through STAT3 inhibitors raises the level of chemoattractant chemokines and enhances tumour cell sensitivity to NK-mediated lysis. All these features, thus make the STAT3 a promising therapeutic target [39,47,48].

Though, the cGAS-STING signalling axis has been identified recently that may require more investigations, various chemotherapeutic as well as radiotherapeutic approaches rely heavily on this signalling pathway to eradicate cancer. For example, etoposide has already been revealed to induce the expression of inflammatory genes, namely IFNβ, IFNA4, and IFI16 [49]. Likewise, another chemotherapeutic agent dimethyloxoxanthenyl acetic acid induces IFNβ and primes CD8^+^ cells in a STING-dependent manner [50]. Furthermore, the Cisplatin treatment has also been proved to activate the cGAS-STING signalling route, it boosts the Type I IFN genes expression, specifically the CXCL9 and CXCL10 genes [51]. These both chemokines have been shown to recruit antigen presenting cells, and T cells to the tumours. Similar to chemotherapeutics, radiotherapy can trigger cGAS-STING signalling axis through the generation of neo-epitopes, which would activate dendritic cells and through the cytoplasmic DNA accumulation directly trigger cGAS-STING in cancer cells. The closely related cGAS, DNA damage, and the immune system offer potential to exploit radiotherapy-induced DNA damage to trigger the immune system to clear more cancer cells [39,52].

## 2. Resistance to Radiation and Chemotherapy by Interferon Signalling Related Proteins

The antitumoral role of IFN has been proven by various studies, for example, murine models showed that IFNα suppresses tumour growth, lack of functional IFNGR (IFNγ receptor) or STAT1 grow tumours faster in presence of carcinogen, and a colon adenocarcinoma model deficient in IFNγ show less tumour regression compared to the control one [9,11,12,53]. On this premise, IFNs have been used as in anti-cancer therapy by conferring pro-apoptotic and anti-proliferative activity to malignant cells, and enhancing the CD8+ T cell content as well as the antigen presentation pathway [54,55]. Paradoxically, IFN response also activates immunosuppressive as well as tumour survival pathways conferring tumour resistance, which lead to poor outcomes [56]. On this regard, IFN based therapies may not be successful at eradicating all cancer cells and those cells that survive frequently arise with more aggressive features; for instance, radiosensitive squamous cell carcinoma (SCC) xenograft becomes resistance after repetitive doses of radiation [57,58,59,60] and repetitive doses of type I IFN confers cell resistance to X-rays [61].

Intrigued by this phenomenon, the scientific community tried to elucidate the mechanisms underlay this effect, and a small group of proteins related to the interferon signalling have been identified in patients that show resistance to radiation and chemotherapy (IRDS genes a subset of ISGs; Figure 2) [61,62]. Clinical data from different breast cancer datasets highlighted a correlation between sensitivity to chemotherapy and low expression of IRDS genes [63], also corroborated in glioblastoma dataset [63]. Additionally, the radiation in breast, prostate, and glioma cancer cells also elevates IRDS gene expression [64]. The repetitive exposure to infrared ray (IR) positively selects the radio-resistant human tumour xenograft Nu61 cells; gene expression analysis reported that 19 of 52 genes differentially expressed were part of the IFN pathway and 25 of them are upregulated by IFN [57,65]. Further investigation in this direction revealed that persistent IFN signalling upregulates the STAT1 gene, which is the key IFN-pathway transducer, but lacks in activating the cytotoxic response [66]. IFN signalling can be continuously activated through unphosphorylated STATs that can bind alternative ISRE (IFN-stimulated response element) sites resulting in the expression of IRDS genes [67,68,69]. A murine model demonstrates that the elevated STAT1 expression leads to radiation-resistance in head and neck cancer xenografts, as well as metastasis and chemoresistance in a melanoma mice model [57,65,70].

Several efforts are being made by the scientific community to understand how the IRDS genes protect malignant cells from eradication. Particularly, the STAT1/IFN pathways are responsive to DNA damage [70,71], and in a healthy state, there is no free DNA or RNA released in the cytosol, whereas upon pathogen attack the exogenous DNA is recognized by the Type I IFN [6,72]. Similarly, the IFN response can be consistently activated by the presence of dsDNA produced by the potent DNA-damage effect caused by chemotherapy [6]. Moreover, there are some arguments as to the source of immunostimulatory DNAs in the DNA damage; Mackenzie et al. suggest lower free dsDNA but rather the micronuclei [73] and Yang et al. emphasises the role of cytoplasmic DNA [37]. Overall, there is a sustained production of low levels of IFN in cancer and chronic inflammation that may cause resistance to DNA damage and facilitate tumour survival [74,75]. All-in-all, these IRDS genes are associated with suppression of T cell toxicity, resistance to DNA damage, metastasis, and epithelial-mesenchymal transition [76] and, thus, proposed as a predictive marker in response to radio- and chemotherapy outcomes.

### 2.1. Biological Pathways, Upstream Regulators and Different IFNs Regulating IRDS Genes

The pathway enrichment analysis combining g:profiler and Cytoscape protocols [78,79,80], assisted to interpret the involvement of IRDS genes [71,74,75] in different biological processes (Figure 2b,c). In total, thirty different biological pathways associated with either of the IRDS genes were classified, and from that 29 pathways were clustered as the ‘interferon viral regulation’ (Figure 2b). The pathway excluded from the ‘interferon viral regulation’ cluster is the ‘negative regulation of endopeptidase activity’, which includes the following genes (Figure 2b); IFI6 (interferon alpha inducible protein 6), THBS1 (Thrombospondin antisense RNA 1), and TIMP3 (Tissue inhibitors of metalloproteinases 3). Seven highly populated biological pathways with the IRDS genes are indentified (abbreviated names); Type I IFN signalling pathway, response to virus, defence response to virus, cellular response to Type I IFN, IFN alpha/beta signalling, response to Type I IFN, and interferon signalling (Figure 2b,c). Surprisingly, a least number of IRDS genes were found in the ‘response to IFN-β’ pathway (Figure 2c). Furthermore, investigating the frequency of a particular IRDS gene occurring in the classified pathways, suggests that IFIT1 (interferon induced protein with tetratricopeptide repeats 1), IFIT3, IFITM1, IRF7 (interferon regulatory factor 7), ISG15 (ISG15 ubiquitin like modifier), MX1 (MX dynamin like GTPase 1), MX2, OAS1 (2’-5’-oligoadenylate synthetase 1), OAS3 and OASL (2’-5’-oligoadenylate synthetase like) genes were found in 20different pathways (Figure 2d and Table 1).

**Figure 3:**
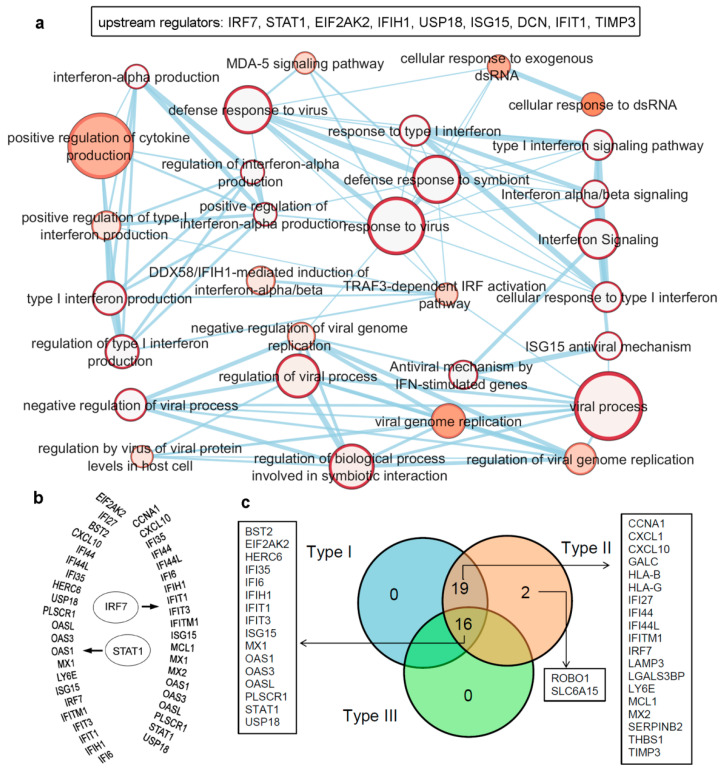
Upstream regulators from the IRDS genes, as well as a list of genes regulated by different IFN types (Type I/II/III). (**a**) IRDS genes (IRF7, STAT1, EIF2AK2, IFIH1, USP18 (ubiquitin specific peptidase 18), ISG15, DCN (decorin), IFIT1 and TIMP3) were found as the upstream regulators in QIAGEN-IPA analysis (Ingenuity Pathway analysis [https://digitalinsights.qiagen.com, accessed on: 7 April 2021]). Genes are presented in ascending order based on the *p*-values, and the bottom panel represents pathway enrichment analysis for upstream regulators performed using g:Profiler [78,79] and Cytoscape package [80]. The node size corresponds to the number of genes in the dataset/gene-set size, and colour of the node corresponds to the number of the geneset for the dataset. Edge size corresponds to the number of genes that overlap between two connected genesets. Intra- and interconnecting nodes means some genes are shared in clusters or pathways, and hence, they are represented as edges. (**b**) Different IRDS genes targeted by two major upstream regulators; IRF7 and STAT1. (**c**) Majority of the IRDS genes were found regulated by both IFNs Type I and II or by all three IFN types, whereas only a small amount of genes are regulated by a single type; Type II IFN. The pie chart is generated considering the experimental datasets from the Interferome database [81].

In order to identify major regulators among the IRDS genes, we performed upstream regulator analysis employing protocols from the ingenuity pathway analysis (QIAGEN-IPA [https://digitalinsights.qiagen.com/, accessed on 7 April 2021]) software (trial version). The data highlighted the following genes as the upstream regulators; IRF7, STAT1, EIF2AK2, IFIH1, USP18 (ubiquitin specific peptidase 18), ISG15, DCN (decorin), IFIT1, and TIMP3 (Figure 3a,b), which are presented in ascending order based on their *p*-values ranking. Furthermore, a set of biological pathways associated with these upstream regulators were classified, as shown in Figure 3a (bottom panel). Particularly, the upstream regulator IRF7 genes (Figure 3b) consists of 33 dataset genes downstream of regulators and 13 other regulators in the network (IRF3, IFNA1/IFNA13, IFN-α and IFNA2 directly connected to IRF7, whereas STAT4, STAT1, STAT3, STAT2, IRF1, MYC (Myc proto-oncogene protein), PML (promyelocytic leukemia) and STAT6 genes are indirectly connected). The causal network analysis in QIAGEN-IPA [https://digitalinsights.qiagen.com/, accessed on 7 April 2021]), suggested IRF7, EIF2AK2, STAT1, USP18, ISG15 and IFIH1 (presented in the ascending order based on their *p*-values) as the master regulator genes.

Majority of the IRDS genes can be found as the subset of ISGs [81,82], and largely these genes are regulated by both Type I and Type II IFNs or by all three IFN types. Therefore, to identify specific IRDS regulated by a single or multiple type IFNs, we performed an extensive search to differentiate these genes using the Interferome database (which consist of datasets to annotated IRGs; IFN-regulated genes) [81]. Analysing the experimental datasets from the interferome [81], revealed that ROBO1 (roundabout guidance receptor 1) and SLC6A15 (solute carrier family 6 member 15) genes are only regulated by single IFN; Type II (Figure 3c). Whereas, 19 genes (CCNA1, cyclin A1; CXCL1; CXCL10; GALC, galactosylceramidase; HLA-B, HLA class I histocompatibility antigen; HLA-G; IFI27; IFI44; IFI44L; IFITM1; IRF7; LAMP3; LGALS3BP, galectin 3 binding protein; LY6E, lymphocyte antigen 6 family member E; MCL1, myeloid cell leukemia 1; MX2; SERPINB2; THBS1; and TIMP3) were regulated by Type I and Type II IFNs, and 16 genes (BST2, EIF2AK2, HERC6 (potential Ubiquitin ligase), IFI35, IFI6, IFIH1, IFIT1, IFIT3, ISG15, MX1, OAS1, OAS3, OASL, PLSCR1, STAT1, and USP18) were found regulated by all three IFN types (Figure 3c).

### 2.2. Functional Interfaces of IRDS Proteins with Different Biomolecules

These IRDS genes are identified to be induced in diverse cancer cell lines in response to chemo- and radiotherapy or mediate experimental resistance [57], as well as signatures have been traced in cancer patients samples that correlates with the resistance [75,83,84]. Therefore, it suggests that targeting or suppressing these IRDS genes in cancer could benefit in increasing the (chemo- and radiotherapy) sensitivity, and few studies are already been performed in this direction: (i) STAT1 is proposed as the main driver of the IRDS expression and resistance, and its overexpression in a SCC cell line conferred resistance to irradiation, whereas its suppression resulted in increased sensitivity [85]. (ii) In breast cancer, IRDS expression measured suggests seven genes (STAT1; MX1; ISG15; OAS1; IFIT1; IFIT3; and IFI44, interferon induced protein 44) whose cancers are resistant to therapies. Silencing of these genes resensitizes triple negative breast cancer (TNBC) cells to chemo- and radiotherapy in vitro and in vivo, illustrating the potential therapeutic power of modulating this response [86].

While interferon responses exert antiviral properties, it is worth noting that they also have roles in the progression of various non-viral diseases. For example, the OAS proteins play as an immune modulator and their level is strongly related to chronic infections, autoimmune disorders, cancers, and infectious diseases. Thus far, they have been overlooked as drug targets due to which there were no molecules to inhibit their activity, besides very recent study put forward the molecules competing the ATP binding site of OAS [87]. Transcription factors activate transcription of their target genes upon binding to different short DNA consensus motifs, and for example, the decoy oligonucleotides comprising such motifs can interact with the DNA binding regions in the transcription factors, thereby blocking their activity. Decoy oligonucleotides have been revealed to trigger the cells death in which the STAT3 gene is activated, and this specifies another possible target for inhibitory molecules in the form of DNA-binding sites [88]. Furthermore, concerning the viral strategies, it also involves targeting the DNA or RNA or nucleotide binding sites of the IRDS genes. For example, the Kaposi’s sarcoma-associated herpesvirus (KSHV), viral homologs of the cellular IFN regulatory factors 3 (vIRF3) selectively bind the DNA binding domain of the IRF7 protein, which leads to the inhibition of IRF7-DNA binding activity and accordingly, suppression of IFNα production as well as IFN-mediated immunity [89].

**Figure 4:**
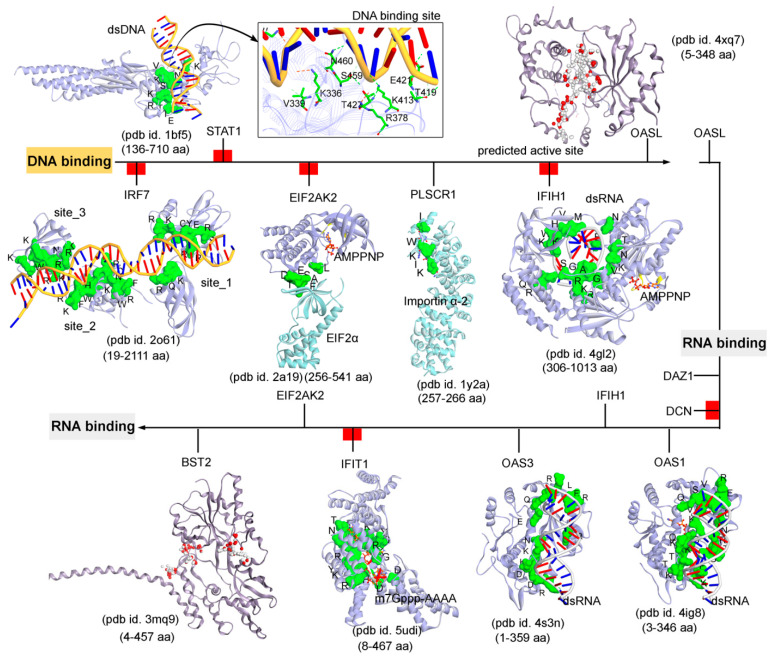
The functional binding sites for a set of IRDS genes with the DNA or RNA molecules. The crystal structures or experimentally derived complexes for the following IRDS genes from the PDB database [7] were analysed; STAT1 (pdb id. 1bf5 [91]), OASL (2’-5’-oligoadenylate synthetase like; pdb id. 4xq7 [92]), IRF7 (interferon regulatory factor 7; pdb id. 2o61 [93]), EIF2AK2 (Eukaryotic Translation Initiation Factor 2 Alpha Kinase 2; pdb id. 2a19 [94]), PLSCR1 (Phospholipid scramblase 1; pdb id. 1y2a [95]), IFIH1 (interferon-induced helicase C-domain-containing protein 1; pdb id. 4gl2 [96]), BST2 (bone marrow stromal cell antigen 2; pdb id. 3mq9 [97]), IFIT1 (interferon induced protein with tetratricopeptide repeats 1; pdb id. 5udi [98]), OAS3 (pdb id. 4s3n [99]), and OAS1 (pdb id. 4ig8 [100]). These DNA or RNA binding interfaces in IRDS genes were defined based on the amino acids involved in the hydrogen bond (h-bond, distance ≥ 3.5 Å) as well as in the pi-stacking (distance ≥ 5 Å) interactions. In addition, the upstream regulators are marked with red label (Figure 3a); IRF7, STAT1, EIF2AK2, IFIH1, DCN and IFIT1, differentiating them with the functional proteins (PLSCR1, OASL, OAS3, BST2 and DAZ1). Interacting residues of IRDS genes with its respective partner are presented in green surface view, with amino acids labeled in black. The DNA and RNA structures are coloured in orange and grey, respectively. For OASL and BST2, the alpha spheres (MOE; Chemical Computing Group Inc., Montreal, QC, Canada) are presented as either “hydrophobic” or “hydrophilic (for lone pair active; LPA)” in red and white colour. Visualization and representation of the protein tertiary structures in this figure, and tracing hydrogen bonds or pi interactions between two partners was performed using the Molecular Operating Environment (MOE; Chemical Computing Group Inc., Montreal, QC, Canada) and BIOVIA Discovery Studio Visualizer (Dassault Systèmes, BIOVIA, San Diego, CA, USA) software programs.

**Figure 5:**
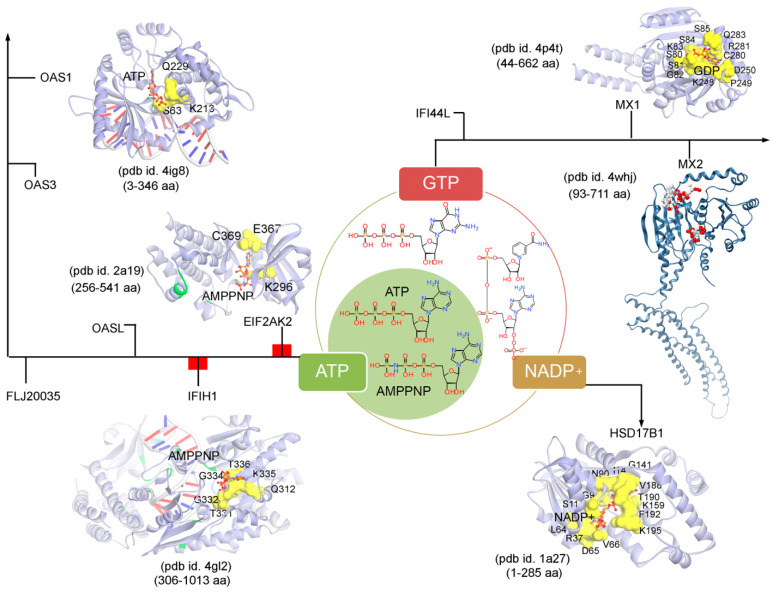
The ATP (adenosine triphosphate), GTP (guanosine triphosphate), or NADP (nicotinamide adenine dinucleotide phosphate) binding residues from a set of IRDS genes. Considering the known tertiary structures from the PDB database [7], the binding sites were defined for the following proteins; OAS1 (pdb id. 4ig8 [100]), EIF2AK2 (pdb id. 2a19 [94]), IFIH1 (pdb id. 4gl2 [96]), MX1 (pdb id. 4p4t [101]), MX2 (pdb id. 4whj [102]), and HSD17B1 (pdb id.1a27 [103]). The ATP, GTP or NADP interfaces were defined considering the amino acids involved in the hydrogen bond (h-bond, distance ≥ 3.5 Å) as well as in the pi-stacking (distance ≥ 5 Å) interactions. The upstream regulators EIF2AK2 and IFIH1 are marked in red label (Figure 3a), differentiating them with the other functional protein. Interacting residues of IRDS genes with its respective partner are presented in yellow surface view, with amino acids labelled in black. The ATP/GTP/NADP and dsRNA structures are coloured in orange and grey, respectively. Particularly for the MX2 gene, the active sites were predicted using the MOE program (Chemical Computing Group Inc., Montreal, QC, Canada), the alpha spheres are presented as either “hydrophobic” or “hydrophilic (for lone pair active; LPA)” in red and white colour. Visualization and representation of the protein tertiary structures for this figure, and tracing hydrogen bonds or pi interactions between two partners was performed using the MOE (Chemical Computing Group Inc., Montreal, QC, Canada) and BIOVIA Discovery Studio Visualizer (Dassault Systèmes, BIOVIA, San Diego, CA, USA) programs.

Subsequently, targeting the IRDS protein interfaces responsible for interacting DNA or RNA or nucleotides by different means may significantly modulate the IRDS response. Functional binding regions in proteins can be identified by traditional experimental approaches such as; X-ray, NMR (nuclear magnetic resonance), cryoEM, CLMS (cross-linking mass spectrometry), etc. [90]. Such identified active site or amino acid hotspots in a protein can be of great importance, which supports the development of different screening platforms that may interfere with crucial recognition processes or other related studies (e.g., mutagenesis). Particularly for the high-throughput screening or in silico SBVS (structure-based virtual screen) of compounds against a protein target, a predefined active site is always effective to classify target specific molecules, as well as can reduce the computational time for the in silico SBVS approach. Considering the importance of such active amino acids, herein, we reported different possible functional active sites of IRDS genes based on their available tertiary structures in the PDB database [7] or proposed them by homology modelling approach (Molecular Operating Environment, MOE; Chemical Computing Group Inc., Montreal, QC, Canada). IRDS genes were distinguished as the DNA or RNA binders, and those essential for binding to nucleotide ATP/GTP/NADP molecules. Particularly, the DNA/RNA-binding functional regions in the structures of the STAT1 [91], OASL [92], IRF7 [93], EIF2AK2 (Eukaryotic Translation Initiation Factor 2 Alpha Kinase 2; or PKR, Protein kinase R) [94], PLSCR1 (phospholipid scramblase 1) [95], IFIH1 (Interferon-induced helicase C-domain-containing protein 1; or MDA5, melanoma differentiation-associated protein 5) [96], BST2 (bone marrow stromal cell antigen 2) [97], IFIT1 [98], OAS3 [99], and OAS1 [100] genes were highlighted (Figure 4). In addition, Figure 5 describes the ATP/GTP/NADP binding amino acids from the OAS1 [100], EIF2AK2 [94], IFIH1 [96], MX1 [101], MX2 [102], and HSD17B1 (hydroxysteroid 17-beta dehydrogenase 1) [103] genes. Certain residues defining the functional active site interfaces or hotspot in these IRDS protein tertiary structures, are listed in Table 2.

The DNA or RNA binding interfaces or active sites in IRDS genes were defined based on the amino acids involved in the hydrogen bond (h-bond, distance ≥ 3.5 Å) as well as in the pi-stacking (distance ≥ 5 Å) interactions. Among different dsDNA binders, the STAT1 protein (pdb id. 1bf5 [91]) formed a conserved active site or pharmacophore model with dsDNA, whereas IRF7 (pdb id. 2o61 [93]) constitute three dsDNA binding sites (Figure 4). Particularly, for the OAS1 (pdb id. 4ig8 [100]), OAS3 (pdb id. 4s3n [99]), and IFIH1 (pdb id. 4gl2 [96]) genes a well-defined binding site with the dsRNA was observed, whereas IFIT1 (pdb id. 5udi [98]) shared a functional interface with the m7Gppp-AAAA molecules (Figure 4). Genes such as the OASL (pdb id. 4xq7 [92]) and BST2 (pdb id. 3mq9 [97]) lacking the binding partners in the structures retrieved from the PDB database [7], their active sites were predicted using the homology modelling module implemented in the MOE program (Chemical Computing Group Inc., Montreal, QC, Canada) (Figure 4 and Table 2). Using the ‘Alpha Shapes’ construction geometric method, the MOE modules (Chemical Computing Group Inc., Montreal, QC, Canada) allows the computation of the binding site of a protein or receptor. This method classifies the alpha spheres as either “hydrophobic” or “hydrophilic (for lone pair active; LPA)”, depending on whether the sphere is in a good hydrogen bonding spot in the receptor. Hydrophilic spheres not near a hydrophobic sphere are eliminated, since these generally correspond to water sites, and sites that are “too exposed” to solvent are filtered out (unlikely to be a good active site). The ‘Apha Shapes’ method in MOE (Chemical Computing Group Inc., Montreal, QC, Canada) identified the largest cluster or active sites residues from each protein are shown in Figure 4.

Differentiating IRDS genes as the ATP/GTP/NADP binders, suggest that OAS1, EIF2AK2, and IFIH1 interact with the ATP or its analogue (AMPPNP, adenylyl imidodiphosphate), MX1 binds with the GDP (guanosine diphosphate), and HSD17B1 binds to NADP+ (Figure 5). A closer view of the ATP/AMPPNP-OAS1 (pdb id. 4ig8 [100]) complex, demonstrated that amino acids mainly interact with the phosphate group of the ATP molecule. Whereas, amino acids from the EIF2AK2 (pdb id. 2a19 [94]) and IFIH1 (pdb id. 4gl2 [96]) genes formed hydrogen bonds with both ends of the AMPPNP molecules, i.e., with the phosphate group as well as with the adenine (nitrogenous base; Figure 5 and Table 2). The lysine (Lys) amino acid was found common in all three genes (OAS1, EIF2AK2 and IFIH1) binding with the phosphate groups of the ATP/AMPPNP molecule. The MX1 (pdb id. 4whj [102]) gene binds with all functional groups (guanine, ribose sugar and phosphate) of the GDP molecule, and similarly, the HSD17B1 (pdb id. 1a27 [103]) interacts to the functional groups from adenine, both ribose and phosphate groups, and nicotinamide (Figure 5). Comparing the pharmacophore designed for the respective IRDS proteins based on the hydrogen bond and pi interactions with ATP/GTP/NADP, suggest that GDP and NADP+ binders formed a conserved active-site hotspots (Figure 5 and Table 2).

Overall, IRDS gene set consist of regulators (e.g., IRF7 and STAT1; Figure 3 and Figure 4), as well as the functional proteins (e.g., OAS1/3/L enzymes; Figure 3, Figure 4 and Figure 5). The regulator IRF7 gene identified in the context with Epstein–Barr virus (EBV), plays a crucial role in both innate and adaptive immunity as the important/key regulator of the Type I IFN responses. Upon pathogen infection, activates IRF7 phosphorylation and it translocates to the nucleus, where binding to the promoter sites of the target genes (along with co-activators) to active transcription [104,105]. In addition, it has been suggested that the appropriate regulation of IRF7 expression is very crucial for normal IFN-mediated physiological functions [106]. The capacity of IRF7 gene to interact with a wider DNA consensus sequence GAAWNYGAAANY, makes it a potential candidate to regulate different target genes involved in different cellular processes [107]. Furthermore, the phosphorylated STAT1 regulator, from various complexes migrate to the nucleus and binds the ISREs GASs leading to the activation of transcription of several genes involved in antiviral responses comprising ISGs, IFNs, IRFs and STATs [20,21]. Though IRF7 and STAT1 are identified as the regulators, their diversity in transcription kinetics indicates that they are regulated by different pathways [108]. The functional proteins, for example, OAS1, OAS2, OAS3, and OASL (OAS family) are antiviral enzymes that are stimulated by interferon. In the presence of double-stranded (ds) RNA viruses these OAS proteins are activated to detect and restrict viral replication [109]. Delaying translation, these OAS proteins operate as a nucleic acid sensor in a more immediate antiviral restriction pathway [110]. In addition to OAS major role as the immune regulators, sever cellular function have been identified, for example, OAS3 synthesize the dimeric 2-5A-activating RNase L derivative, which is suggested as inhibitory molecules against breast cancer growth [111].

### 2.3. Diverse Set of Strategies Practiced Targeting IRDS Genes

IRDS genes have been proposed as a predictive marker of response to radio- and adjuvant chemotherapy, and therefore, targeting IRDS mediated resistance could provide an effective approach to resensitize tumours cells, and improve the outcome of anti-cancer treatment. Herein, we reviewed different strategies already practiced to modulate the activities of IRDS genes. Particularly, the STAT1 gene has been reported to be the main driver of IRDS expression, and efforts to identify potential inhibition strategies have been primarily focused on direct STAT1 inhibition; disruption of STAT dimerization as well as phosphorylation, and inhibition of STAT-mRNA/DNA binding. For example, Fludarabine, a STAT1 inhibitor approved for the treatment of various haematological malignancies, acts by inhibiting STAT1 phosphorylation in normal and malignant cells, thus leading to downregulation and impaired STAT1 signalling [112].

Currently, combined treatment of Fludarabine with pegylated liposomal doxorubicin is being evaluated (phase 2, clinical trials) in patients with refractory ovarian cancer. Moreover, it has been reported that a group of phosphopeptide mimetics of STAT1, notably ISS-840, have been found to inhibit STAT1 by binding within the SH2 domain and disrupting dimerization [113]. Pravastatin, a synthetic small molecule inhibitor of 3-hydroxy-3-methylglutaryl coenzyme A (HMG-CoA) reductase, reportedly reduces IFNγ activity via modulation of STAT1. In studies of apolipoprotein E-knockout mice fed on a cholesterol-rich diet, treatment with pravastatin decreased serum IFNγ levels by attenuating STAT1 activity [114]. Another interesting strategy is inhibition of the STAT1 DNA-binding domain with the oligodeoxynucleotide (ODN) decoys. Competition between the ODN decoys and promoter sequences for binding of the transcription factors facilitate gene suppression at the transcriptional level [88]. In addition, to such direct targeting of STAT1, there have been several indirect targeting strategies that also constitute an important mechanism of STAT1 inhibition. STAT1 activity can be suppressed by SOCS (suppressor of cytokine signalling) proteins a negative-feedback inhibitor of JAK/STAT pathway, by induction of phosphatases and STAT1 dephosphorylation, as well as by PIAS (protein inhibitors of activated STATs) binding to phosphorylated STAT dimers disrupting DNA binding [115,116,117]. Furthermore, blocking upstream receptors also inhibits STAT1. Recently, the JAK2 inhibitor (SAR302503) role in suppressing STAT1 activation in radioresistant non-small cell lung cancer (NSCLC) cell lines has been recorded [118]. Despite numerous studies on the modulation of STAT proteins, and several discovered and developed inhibition strategies only a few inhibitors entered clinical trials.

Upregulated IRF7 gene in breast cancer elicits an immune response that is strongly associated with cancer cells decreased growth, nevertheless, also contributes to cell resistance to chemotherapy [119]. Currently, the only IRF inhibition methods reported are based on IRFs indirect modulation. Direct inhibition strategy has not been widely investigated, and the only direct approach applied to inhibit IRF employ siRNA and miRNA to target IRFs transcription. The IRF-DNA binding site has been proposed as the promising active site for inhibition, which could provide a new opportunity for therapeutics [89]. In addition, it has been demonstrated that ORF45 (Open Reading Frame 45) of KSHV acts as IRF7 phosphorylation inhibitor, which blocks the nuclear translocation of the IRF7 gene [120].

Against the PKR or EIF2AK2 gene, the imidazolo-oxindole PKR inhibitor C16 is the most broadly used pharmacological drug. It is a highly robust and selective small-molecule inhibitor, which binds the ATP binding site of PKR and blocks RNA-induced PKR autophosphorylation [121]. The 2′-5′ oligoadenylate synthetase family (OAS1, OAS2, OAS3, and OASL) consists of antiviral enzymes stimulated by interferon [109]. OAS has been associated with immune-regulatory functions that facilitate infectious diseases, autoimmune disorders, chronic inflammatory conditions, and cancer [122,123]. Despite this, neither OAS enzymes have not been pursued as drug targets, nor direct OAS inhibitory strategies have been reported. Recently, an in silico study identified 37 molecules that could compete for ATP binding sites of OAS proteins and inhibit their enzymatic activity [87], however, these findings require further experimental validation. In addition, the demethylating drugs could exert their chemotherapeutic effect by hyperactivating RNA sensing pathways and downstream toxicity [124]. Another member of IRDS genes, PLSCR1, belongs to a conserved family of genes PLSCR, and it possesses a conserved calcium ion binding domain, DNA-binding domain and transmembrane region [125]. In studies, PLSCR1 has been blocked with direct inhibition methods, notably with siRNA or by knocking down PLSCR1 with small hairpin RNA (shRNA) [126].

Therapeutic targeting IFIH1 or MDA5 gene is primary focused on identification of potent nucleic acid agonists, as MDA5 is one of a pivotal sensors of pathogen-associated molecular pattern (PAMP) and mediates downstream signalling to activate an antiviral and immunomodulatory response [127]. Recently, two high molecular weight dsRNA derived from natural sources have been reported; dsRNA (rb-dsRNA) corresponding to the genome of endornavirus extracted from rice bran [128] and nucleic acid band 2 (NAB2), a dsRNA corresponding to a yeast virus of the Totiviridiae family [129]. The specificity of RNA binding site in IFIT1 protein has been reported to be modulated by binding of another IRDS gene IFIT3. IFIT3 binding extends the half-life of IFIT1 and allosterically regulates the IFIT1-RNA binding channel, and thus, manipulating the protein stability [130]. The study covering the role of IFIT1 and IFIT3 genes on drug resistance in OSCC cells (oral squamous cell carcinoma) showed that silencing of IFIT1 and IFIT3 by shRNA increased the sensitivity to cisplatin, thus further implicating their role in chemotherapy resistance [131].

The BST2 also known as Tetherin have been suggested to play a role in the growth and progression in various cancers. Recent reports implicate BST2 elicits its pro-tumor effects through the formation of BST2 dimers. Mahauad-Fernandez et al., selected BST2 extracellular domain as potential target for drug treatment [132]. They generated small peptides (B49) with a specific binding to the BST2 extracellular domain, thus disrupting the dimerization of BST2 and inhibiting BST2 mediated tumorigenesis and metastasis. The CXCL1 (C-X-C motif chemokine ligand 1) and CXCL10, ligands for chemokine receptor 2 (CXCR2) and chemokine receptor 3 (CXCR3), respectively, are the only members of CXC chemokines family identified as IRDS genes and have been implicated in cancer progression and metastasis. CXCLs/CXCR axis is an attractive target for drug treatment, however, recent strategies have been primarily focused on receptor inhibition. Nonetheless, targeting the CXCR ligands also showed encouraging results in preclinical studies. Numerous methods based on CXCLs inhibition have been developed including application of neutralizing antibodies, targeting molecules in the upstream signalling pathway or miRNA transfection [133].

## 3. Viruses and IRDS Genes

Evolution of the innate and adaptive immunity was shaped by species-specific viruses, and many elements of these networks are brought by viruses themselves [134,135,136]. The ability of retroviruses to integrate to the host DNA, expand and reintegrate allows for dispersing their sequences all over the genome. These retroviral sequences play essential regulatory roles in immune response, for example, IFNγ network is controlled by lineage-specific endogenous retroviruses (ERVs) scattered independently in diverse mammalian genomes as IFN-inducible enhancers [137]. They constitute the binding sites for IFN-induced transcription factors STAT1 and IRF1, allowing for expression of ISGs. It has been often observed that persistent viruses protect the host from infections of similar viruses. Any new colonizers need to outsmart the encountered immunity mechanisms and introduce new ones, and thus, enhancing immunity of the host. Accordingly, viruses have developed various ways to evade the antiviral responses; they either inhibit the production of IFNs, block inter- and intra-cellular IFNs signalling, or impair the action of IFN-induced antiviral proteins. Depending on the strategy of a particular virus and the immune status of the host, viruses not only impair but can utilize IFN signalling to establish a stable and inapparent infection in their hosts.

STAT1 is inhibited by several viruses applying different strategies, for example, the V protein of simian virus 5 (SV5) and the C protein of the Sendai virus directly target STAT1 for proteasomal degradation [138,139]. Instead, E1A of adenovirus directly binds and inactivates STAT1 [140], while vaccinia virus phosphatase VH1 (Vaccinia virus gene H1) binds and dephosphorylates STAT1 [141]. Although, the STAT1 degrading IFN-insensitive SV5 virus continues to produce its proteins, the IFN-sensitive variant has an impaired protein synthesis, thus can hide from T cells or antibody-mediated recognition. The IFN resistant SV5 is a more acute variant, inducing strong T cell and B cell responses, while the IFN-sensitive can hide and persist. This is in line with the RNA quasispecies concept, where RNA viruses due to the infidelity of the RNA polymerase, form a population of diverse variants of both collaborative and interfering actions [142,143,144,145,146]. This diversity allows the virus to adjust to the changing environments, and to the immune status of the host. Interestingly, DNA viruses use non-coding RNAs like microRNAs to fine-tune the host immune response, e.g., EBV-encoded miR-BART20-5p and miR-BART8 inhibit the IFNγ-STAT1 pathway associated with progression of nasal NK-cell lymphoma [147].

IFN signalling and viral dsRNA lead to the activation of the OAS/RNase L system, which results in the cleavage of viral and cellular ssRNA. OSL family members OAS1, OAS2 and OAS3 activated by dsRNA binding, synthesize short oligonucleotide secondary messengers (2-5A) that induce dimerization and activation of otherwise latent RNase L. Activated RNase L, either directly cleaves viral RNA or cellular ssRNA, which boosts IFN signalling and induces cell apoptosis. It seems that the most efficient way would be to directly inhibit the RNase L activity, as does the Theiler’s murine encephalomyelitis virus (TMEV) protein L* [148] or to escape from the RNase L cleavage via formation of cleavage resistant secondary structures (poliovirus [149]) or via decreasing the number of cleavage sites (Hepatitis C virus genotype 1 [150]). However, many viruses act upstream of the effector by; (i) sequestering dsRNA and preventing OAS activation (Influenza A virus NS1 [151], vaccinia virus (VV) E3L [152], the σ3 outer capsid protein of reoviruses [153], Tar protein of HIV [154]), (ii) expression of viral mRNA decapping enzymes to limit dsRNA accumulation [155,156], (iii) degrading 2-5A messengers by a viral phosphodiesterase (ns2 protein of mouse hepatitis virus (MHV) [157], C-terminal domain of rotavirus protein VP3 [158,159]), and (iv) production of inactive or inhibitory 2-5A (herpes simplex virus HSV-1 and HSV-2 [160]).

The fact that viruses inhibit steps upstream of RNase L activation, indicates that they might have RNase L-independent antiviral effects. Indeed, upon dsRNA binding, OAS proteins activate cellular helicases like RIG-1 (retinoic acid inducible gene 1) and MDA5 to potentiate interferon pathways. MDA5, binds long dsRNA (>1000 bp) with no end specificity [161,162], which causes its oligomerization and leads to the association with the adaptor molecule MAVS (mitochondrial antiviral signalling protein) and activation of downstream signalling cascades inducing transcription of IFNs and ISGs [96,163,164]. Some of the known mechanisms used by viruses to disrupt recognition by MDA5 are: (i) sequestration of viral RNA PAMPs, e.g., Ebola virus (EBOV) and Marburg virus (MARV) VP35 proteins bind viral dsRNA to prevent their recognition [165,166], (ii) direct binding to RIG-I like receptors (RLRs), e.g., all viruses from paramyxovirinae subfamily encode a protein V that can bind to MDA5 and disrupt downstream signalling responsible for IFN induction [85,167], and (iii) regulation of phosphorylation events, e.g.,Nipah virus (NiV) and measles virus (MeV) encode V proteins that can bind to PP1α/γ (protein phosphatase 1) and inhibit the dephosphorylation of MDA5 at Ser88 and consequently activating the MDA5 gene [168,169].

Interestingly, viruses can also make use of the MDA5 to promote disease, which has been recently proved in pathogenesis of vitiligo under virus invasion. Virus invasion significantly activated MDA5 as well as promoted the secretion of CXCL10 and CXCL16 in keratinocytes, which are the two vital chemokines for the cutaneous infiltration of CD8+ T cells in vitiligo. Under virus invasion, IFNβ mediated by the MDA5-MAVS-NF-κB/IRF3 signalling pathway, induced the secretion of CXCL10 via the JAK/STAT1 pathway and MDA5-mediated IRF3 transcriptionally activated the production of CXCL16 in keratinocytes [170]. Furthermore, the IFITMs are restriction factors conferring potent antiviral activity to the cell host, and IFITM1/2/3 proteins inhibit the replication of a wide range of viruses. Individually, the IFITM1 gene is able to attenuate different types of viruses such as SARS (Severe Acute Respiratory Syndrome) coronavirus, Influenza A, and HCV [171,172,173,174].

IFIT1, can primarily interfere with the interaction of eIF4E with the cap structure, and competes with eIF4E and eIF4F for binding to cap 0 mRNA [175,176,177] or suppress an internal ribosome entry site (IRES)-dependent translation by targeting eIF3-dependent steps in the viral RNA translation initiation process, in the case of hepatitis C virus [178,179]. IFIT1 can also recognize viral RNA that carries a triphosphate group on its 5′ terminus (PPP-RNA), and sequesters it from the actively replicating pool [180]. As IFIT1 is one of the most strongly induced genes by the cell-intrinsic innate immune responses, pathogenic viruses have evolved specific and efficient mechanisms to overcome its inhibitory action, such as their own capping machinery for N-7 and 2′-O methylation of viral RNA [178], ‘‘cap snatching’’ mechanism, which refers to ‘stealing’ cap structures from cellular mRNA [181,182] or using host methyltransferases in the nucleus to generate cap 1 structures on 5′ end [183]. Viruses are also using cap-independent translation or covalent binding of viral proteins to the 5′-end of the RNA. Interestingly, in the case of alphaviruses, the viral replication varies in the presence of IFIT1, depending on the structures of the 5’-untranslated regions (UTRs) in their genomes. They are using a stable stem-loop structure in their 5′-UTR to antagonize the IFIT1 binding and antiviral activity. Additionally, in viruses IFIT1 acts as an antiviral effector molecule as well as an inducer of innate immunity, since the presence of IFIT1 at higher levels makes IFIT1-resistant wild-type alphaviruses more potent inducers of Type I IFN [184].

In vitro studies have elaborated that tumour-associated human papillomavirus (HPVs) oncoproteins utilize various strategies to interfere with the IFN-receptor pathways. By binding with the TYK2, they hamper phosphorylation of the transcription factors, STAT1 and STAT2 that are required for IFN-stimulated gene transcription [185,186]. High-risk HPV oncoprotein E6 directly impairs STAT1 transcription and translation [187,188,189], while E7 protein form interaction with the IRF9 gene, and thus, preventing the IRF9 binding with phosphorylated STAT1 and STAT2, which forms a complex for nucleus translocation [190,191]. This perhaps explains the limited effect of IFN therapy in the treatment of HPV genital infections highlighted during the past few years [192]. It is of interest to note that the response rate to IFNα in low-risk HPV infection patients is higher compared to those with high-risk HPV infection [193]. Furthermore, cervical cancer patients’ biopsy samples showed downregulation of Type I IFN expression compared to tissue from normal individuals [194], which implies that high-risk HPVs more efficiently promote host resistance to IFN signalling [186].

## 4. Conclusions and Outlook

The mechanisms by which IFNs promote the efficacy of standard-of-care cancer treatments, remains an area of active investigation. According to antiviral potency, several studies ranked the IFN subtypes, with IFNα8 often being the most potent and IFNα1 up to 1000-fold less potent. These IFNα offers a significant benefit in terms of overall survival when given as an adjuvant therapy to surgery in high-risk patients with malignant melanoma, sequentially with dacarbazine. Radiotherapy and chemotherapy are the standard treatments for beating cancer, but tumour resistance to these methods is rising a great concern. Interferon-stimulated genes expression encompass an IFN-related DNA damage resistance signature or IRDS, which is strongly associated with resistance to radiation and chemotherapy across different tumours. An overview of different strategies adopted to suppress these IRDS genes (for example; STAT1, IRF7, OAS family and BST2) in cancer inducing the sensitivity (chemo- and radiotherapy), as well as different ways by which the viruses inhibit the IRDS genes are addressed in this review. In addition, major upstream regulators (IRF7, STAT1, EIF2AK2, IFIH1, USP18, ISG15, DCN, IFIT1 and TIMP3) from the IRDS gene set were identified, and different IFNs regulating these genes were outlined. Both Type I and Type II IFNs regulate 19 IRDS genes and 16 genes were found regulated by all three IFN types, whereas ROBO1 and SLC6A15 genes are only regulated by Type II IFN.

The pathway enrichment data revealed association of IRDS genes with different significant biological pathways, among which majority of them are connected to the ‘interferon viral regulation’, excluding the ‘negative regulation of endopeptidase activity’ which carries IFI6, THBS1 and TIMP3 genes. Additionally, seven highly populated biological pathways with the IRDS genes were identified, and with recording their frequently associated genes (IFIT1/3, IFITM1, IRF7, ISG15, MX1/2 and OAS1/3/L). To capture novel insights within IRDS genes we further investigated their tertiary structures, with providing a glance of functional interfaces. Based on the known structures coding IRDS genes, the hotspots regions making interactions with DNA/RNA and ATP/GTP/NADP were defined, using an established computational workflow. Alike the STAT1 and IRF7 proteins forming a well-defined pharmacophore active sites with dsDNA, the genes OAS1, OAS3, and IFIH1 defined their pharmacophore with dsRNA. The Lys residue was found common in all three genes (OAS1, EIF2AK2 and IFIH1) binding with the phosphate groups of the ATP molecule. Additionally, the MX1 and HSD17B1 genes defined a conserved active site model with the GDP and NADP+ molecules, respectively. These details revealing the IRDS genes can be of immense importance, as a predefined active site is always effective to classify target specific molecules. In particular, structural knowledge can support different strategies to target the identified functional sites in IRDS genes, and may open doors for several genes that have notfundamentally validated for such applications.

## Figures and Tables

**Table 1:** Dataset retrieved from the pathway enrichment analysis using the g:profiler and Cytoscape protocols [78,79,80], representing the number of IRDS genes involved in a particular pathway.

Name	Genes	*GS_DESCR	*gs_Size
GO:0043903	ISG15|STAT1|IFI27|MX1|OAS1|OASL|IFIT1|OAS3|IFITM1	regulation of symbiotic process	9
REAC:R-HSA-909733	IFI27|USP18|OAS1|HLA-B|IFIT1|IFI6|OAS3|IFI35|IFIT3|IFITM1|ISG15|MX1|OASL|IRF7|MX2	Interferon alpha/beta signalling	15
GO:0045069	ISG15|IFI27|MX1|OAS1|OASL|IFIT1|OAS3|IFITM1	regulation of viral genome replication	8
GO:0009615	STAT1|IFI27|OAS1|IFIT1|IFI6|OAS3|IFIT3|IFITM1|ISG15|MX1|IFI44|OASL|IFIH1|IFI44L|IRF7|MX2	response to virus	16
GO:1903900	ISG15|IFI27|MX1|OAS1|OASL|IFIT1|OAS3|IFITM1	regulation of viral life cycle	8
GO:0060700	OAS1|OASL|OAS3	regulation of ribonuclease activity	3
GO:1903901	ISG15|MX1|OAS1|OASL|IFIT1|OAS3|IFITM1	negative regulation of viral life cycle	7
GO:0032727	STAT1|IFIH1|IRF7	positive regulation of interferon-alpha production	3
REAC:R-HSA-8983711	OAS1|OASL|OAS3	OAS antiviral response	3
GO:0050792	ISG15|STAT1|IFI27|MX1|OAS1|OASL|IFIT1|OAS3|IFITM1	regulation of viral process	9
GO:0032069	OAS1|OASL|OAS3	regulation of nuclease activity	3
GO:0016032	ISG15|STAT1|IFI27|MX1|OAS1|OASL|IFIT1|HLA-B|IFIH1|OAS3|IRF7|IFITM1	viral process	12
GO:0010951	TIMP3|IFI6|THBS1	negative regulation of endopeptidase activity	3
GO:0051607	STAT1|IFI27|OAS1|IFIT1|IFI6|OAS3|IFIT3|IFITM1|ISG15|MX1|OASL|IFIH1|IFI44L|IRF7|MX2	defense response to virus	15
GO:0071357	STAT1|IFI27|USP18|OAS1|HLA-B|IFIT1|IFI6|OAS3|IFI35|IFIT3|IFITM1|ISG15|MX1|OASL|IRF7|MX2	cellular response to type I interferon	16
GO:0035455	MX2|IFIT3|IFITM1	response to interferon-alpha	3
GO:0035456	STAT1|IFITM1	response to interferon-beta	2
GO:0045071	ISG15|MX1|OAS1|OASL|IFIT1|OAS3|IFITM1	negative regulation of viral genome replication	7
REAC:R-HSA-913531	STAT1|IFI27|USP18|OAS1|HLA-B|IFIT1|IFI6|OAS3|IFI35|IFIT3|IFITM1|ISG15|MX1|OASL|IRF7|MX2	Interferon Signalling	16
REAC:R-HSA-877300	OAS1|HLA-B|OASL|OAS3|IRF7	Interferon gamma signalling	5
GO:0060333	STAT1|OAS1|HLA-B|OASL|OAS3|IRF7	interferon-gamma-mediated signalling pathway	6
REAC:R-HSA-1169408	ISG15|STAT1|USP18|MX1|IFIT1|MX2	ISG15 antiviral mechanism	6
GO:0034341	STAT1|OAS1|HLA-B|OASL|OAS3|IRF7|IFITM1	response to interferon-gamma	7
GO:0034340	STAT1|IFI27|USP18|OAS1|HLA-B|IFIT1|IFI6|OAS3|IFI35|IFIT3|IFITM1|ISG15|MX1|OASL|IRF7|MX2	response to type I interferon	16
GO:0048525	ISG15|STAT1|MX1|OAS1|OASL|IFIT1|OAS3|IFITM1	negative regulation of viral process	8
GO:0060337	STAT1|IFI27|USP18|OAS1|HLA-B|IFIT1|IFI6|OAS3|IFI35|IFIT3|IFITM1|ISG15|MX1|OASL|IRF7|MX2	type I interferon signalling pathway	16
GO:0019058	ISG15|IFI27|MX1|OAS1|OASL|IFIT1|OAS3|IFITM1	viral life cycle	8
GO:0019079	ISG15|IFI27|MX1|OAS1|OASL|IFIT1|OAS3|IFITM1	viral genome replication	8
GO:0071346	STAT1|OAS1|HLA-B|OASL|OAS3|IRF7	cellular response to interferon-gamma	6
REAC:R-HSA-1169410	ISG15|STAT1|USP18|MX1|OAS1|OASL|IFIT1|OAS3|MX2	Antiviral mechanism by IFN-stimulated genes	9

*GS_DESCR, the gene set description; gs_szie, gene set size.

**Table 2:** Residues defining the pharmacohpore model for STAT1, PLSCR1, BST2, EIF2AK2, HSD17B1, IFIH1, IFIT1, IRF7, MX1, MX2, OAS1, OAS3 and OASL genes, generated from the crystal structures or predicted using the homology modelling approach using the MOE (Chemical Computing Group Inc., Montreal, QC, Canada) package.

Gene	pdb id.	Resolution (Å), Residue Range	Binding Partner	Binding Residues or *(Active Site Predicted)
STAT1	1bf5 [91]	2.90, 136–710	dsDNA	K336, R378, K567, T419, E421, K413, T427, S459, N460, V339, R378
IRF7	2o61 [93]	2.80, 19–2111	dsDNA	site #1 = R33, R35, Y36, C38, E39, R41, S42, K122, K123, R124, K218, K221, R246, Q247; site #2 = F1014, W1044, H1046, F1047, R1049, K1050, W1063, R1067, K1092, N1094, R1096, C1097, R1100, K1120; site #3 = W2038, G2041, K2077, R2078, N2079, R2081, R2086, K2087, K2098, K2105
PLSCR1	1y2a [95]	2.20, 257–266	Importin α-2	K258, I259, S260, K261, W263, I266
BST2	3mq9 [97]	2.80, 4–457	RNA	* (D14, K15, G16, A63, A109, V110, E111, P229, G260, V261, L262, V293, K297, P298, L299, G300, A301, H64, D95, A96, V97, R98, Y99, N100, Y171, G174, K175, Y176, I329, M330, P331, N332, I333, W230, A231, W232, SER233, D65, R66, G68, G69, Y70, P334, GLN335, M336, SER337, W340, F169, I178, K179, A338, I368, T369, A371, R372, Y167, PHE169, K170, E328)
EIF2AK2	2a19 [94]	2.50, 256–541	EIF2α	L452, D486, T487, A488, F489, E490, S492
			AMPNP	K296, E367, C369
HSD17B1	1a27 [103]	1.90, 1–285	NADP+	G9, S11, I14, R37, L64, D65, V66, N90, G141, K159, V188, T190, F192, K195
IFIH1	4gl2 [96]	3.56, 306–1013	dsRNA	N364, K365, V366, T413, N419, E451, Q580, R605, K726, R728, G756, A757,G758, S761, N812, R843, M926, H927, K983, K1001, W1003, V1004
			AMPNP	K335, G334, T336, Q312, T331, G332
IFIT1	5udi [98]	1.58, 8–467	m7Gppp-AAAA	R38, Q42, T48, K151, G154, Y157, R187, G190, N216, Y218, R255, Y256, K259, R262, Q290, H289, K336, D345, D379
MX1	4p4t [101]	2.30, 44–662	GDP	S80, S81, G82, K83, S84, S85, K248, P249, D250, C280, R281, Q283
MX2	4whj [102]	3.20, 93–711	GDP	* (D126, Q127, S128, S129, G130, K131, S132, L135, E136, S139, G140, V141, A142, L143, P144, V150, T151, A166, A168, P183, G184, E187, I213, S215, E217, V218, D225, L226, P227, G228, Q330, R328, G329, E332, I333, T334, N335, R336, L337, S338, L339, K345)
OAS1	4ig8 [100]	2.70, 3–346	dsRNA	K14, E17, P22, T24, R27, N31, D35, K42, E43, R47, V55, S56, V58, K60, G67, Q158, K199, Q200, T203, K204, K206, T247
			ATP	S63, K213, Q229
OAS3	4s3n [99]	2.00, 1–359	dsRNA	D12, R13, R30, R41,E42, R53, L55, T57, Q155, E186, N193, K198, K200, D244
OASL	4xq7 [92]	1.60, 5–348	apo	* (V67, G68, S69, F70, G71, N72, T74, V75, L76, S78, T79, R80, E81, V82, E83, L84, V85, E129, R131, V132, P133, A135, T150, T152, V154, I178, C181, F187, P189, S192, Q195, R196, V199, K214, Y217, Q218, Q219, K222, A223, A228, N229, L230, P231, P232, L233, Y234, E237, C291, K294, Q295, K298, D305, T310, L311, N312, V313, A314, E315, Y317)

* Abbreviations: STAT1 (signal transducer and activator of transcription 1), PLSCR1 (phospholipid scramblase 1), BST2 (bone marrow stromal cell antigen 2), EIF2AK2 (eukaryotic translation initiation factor 2 alpha kinase 2), HSD17B1 (Hydroxysteroid 17-Beta Dehydrogenase 1), IFIH1 (interferon-induced helicase C-domain-containing protein 1), IFIT1 (interferon induced protein with tetratricopeptide repeats 1), IRF7 (interferon regulatory factor 7), MX1 (MX dynamin like GTPase 1), MX2, OAS1(2’-5’-oligoadenylate synthetase 1), OAS3, and OASL (2’-5’-oligoadenylate synthetase like).

## Data Availability

Data is contained within the article.

## Abbreviations

AMP	Adenosine Monophosphate
aa	Amino acids
AMPPNP	Adenylyl Imidodiphosphate
ATP	Adenosine Triphosphate
BST2	Bone Marrow Stromal Cell Antigen 2
CCNA1	Cyclin A1
CD8+ T-cells	Cytolytic T cell
CLMS	Cross-Linking Mass Spectrometry
CIN	Chromosomal Instability
GMP	Cyclic Guanosine Monophosphate
CPD	Cytoplasmic Domain
cryoEM	Cryo-electron Microscopy
CXCL	C-X-C motif Chemokine Ligand
CXCR	Chemokine Receptor
DCs	Dendritic Cells
DCN	Decorin
DDR	DNA damage response
EBOV	Ebola Virus
EBV	Epstein–Barr Virus
EIF2AK2	Eukaryotic Translation Initiation Factor 2 Alpha Kinase 2
eIF3	Eukaryotic Initiation Factor 3
ER	Estrogen Receptor
ERVs	Endogenous Retroviruses
ETD	Extracellular Topological Domain
FDA	Food and Drug Administration
GASs	Gamma-Activated Sequences
GALC	Galactosylceramidase
GBPs	Guanylate Binding Proteins
GDP	Guanosine Diphosphate
GS_DESCR	Gene Set Description
gs_szie	Gene set size
Gtp	Guanosine Triphosphate
HBV	Hepatitis B virus
HCV	Hepatitis C virus
HMG-CoA	3-Hydroxy-3-methylglutaryl Coenzyme A
HSD17B1	Hydroxysteroid 17-Beta Dehydrogenase 1
HSV	Herpes Simplex Virus
HLA	Histocompatibility Antigen
IFI	Interferon Alpha Inducible
IFIH1	Interferon-induced Helicase C-domain-containing protein 1
IFIT	interferon Induced protein with Tetratricopeptide repeats
IFITM	Interferon-induced Transmembrane protein
IFN	Interferon
IFN-I	Type 1 Interferon
IFN-II	Type 2 Interferon
IFN-III	Type 3 Interferon
IFNAR1	Interferon Alpha and Beta Receptor subunit 1
IFNAR2	Interferon Alpha and Beta Receptor subunit 2
IFNGR	Interferon-Gamma Receptor
IFNLR	Interferon Lambda Receptor
IL10Rβ	Interleukin 10 Receptor Beta
IRES	Internal Ribosome Entry Site
IRF	Interferon Regulatory Factor
IRGs	IFN-regulated Genes
IR	Infrared Ray
IRDS	IFN-related DNA Damage resistance Signature
ISRE	IFN-Stimulated Response Element
ISG	IFN-Stimulated Gene
ISG15	ISG15 Ubiquitin Like Modifier
IPA	Ingenuity Pathway Analysis
JAK	Janus Kinase
KSHV	Kaposi’s Sarcoma-Associated Herpesvirus
LPA	Lone Pair Active
LGALS3BP	Galectin 3 Binding Protein
LY6E	Lymphocyte antigen 6 family member E
MARV	Marburg Virus
MAVS	Mitochondrial Antiviral Signalling protein
MCL1	Myeloid Cell Leukemia 1
MDA5	Melanoma Differentiation-Associated Protein 5
MeV	Measles Virus
MHV	Mouse Hepatitis Virus
MOE	Molecular Operating Environment
MX	MX dynamin like GTPase
NAB2	Nucleic Acid Band 2
NADP	Nicotinamide Adenine Dinucleotide Phosphate
NF-κB	Nuclear Factor Kappa-light-chain enhancer of activated B cells
NiV	Nipah Virus
NK	Natural Killer
NMR	Nuclear Magnetic Resonance
NOS2	Nitric Oxide Synthase 2
NS1	Non-Structural Protein 1
NSCLC	Non-Small Cell Lung Cancer
MYC	Myc proto-oncogene protein
OAS	2’-5’-Oligoadenylate Synthetase
OASL	2’-5’-Oligoadenylate Synthetase Like
ODN	Oligodeoxynucleotide
ORF45	Open Reading Frame 45
OSCC	Oral Squamous Cell Carcinoma
P	Phosphate
PAMP	Pathogen-Associated Molecular Pattern
PDB	Protein Data Bank
PEG	Pegylated
PIAS	Protein Inhibitors of Activated Signal Transducer
PKR	Protein Kinase R
PML	Promyelocytic Leukemia
PLSCR1	Phospholipid Scramblase 1
PP1	Protein Phosphatase 1
PPP	Triphosphate
FDR	False Discovery Rate
rb-dsRNA	dsRNA extracted from rice bran
RIG-1	Retinoic Acid Inducible Gene 1
RLRs	RIG-I like receptors
ROBO1	Roundabout Guidance Receptor 1
SARS	Severe Acute Respiratory Syndrome
SBVS	Structure-Based Virtual Screen
SCC	Squamous Cell Carcinoma
shRNA	Small hairpin RNA
SOCS	Suppressor of cytokine signalling
STAT	Signal Transducer and Activator of Transcription
SV5	Simian Virus 5
SLC6A15	Solute Carrier Family 6 Member 15
THBS	Thrombospondin
TIMP	Tissue Inhibitors of Metalloproteinases
TMD	Transmembrane Domain
TMEV	Theiler’s Murine Encephalomyelitis Virus
TNBC	Triple Negative Breast Cancer
TRIM	Tripartite Motif Protein
TYK	Tyrosine Kinase
UTRs	Untranslated Regions
VV	Vaccinia Virus
VH1	Vaccinia virus gene H1
vIRF	Viral homologs of the cellular IFN regulatory factors
USP18	Ubiquitin Specific Peptidase 18

## References

[1] Deng L., Liang H., Fu S., Weichselbaum R.R., Fu Y.X. (2016). From DNA damage to nucleic acid sensing: A strategy to enhanceradiation therapy. Clin. Cancer Res..

[2] Burnette B.C., Liang H., Lee Y., Chlewicki L., Khodarev N.N., Weichselbaum R.R., Fu Y.X., Auh S.L. (2011). The efficacy of radiotherapy relies upon induction of type i interferon-dependent innate and adaptive immunity. Cancer Res..

[3] Lim J.Y., Gerber S.A., Murphy S.P., Lord E.M. (2014). Type I interferons induced by radiation therapy mediate recruitment and effector function of CD8(+) T cells. Cancer Immunol. Immunother..

[4] Sarhan J., Liu B.C., Muendlein H.I., Weindel C.G., Smirnova I., Tang A.Y., Ilyukha V., Sorokin M., Buzdin A., Fitzgerald K.A. (2019). Constitutive interferon signaling maintains critical threshold of MLKL expression to license necroptosis. Cell Death Differ..

[5] Snyder A.G., Hubbard N.W., Messmer M.N., Kofman S.B., Hagan C.E., Orozco S.L., Chiang K., Daniels B.P., Baker D., Oberst A. (2019). Intra tumoral activation of the necroptotic pathway components RIPK1 and RIPK3 potentiates antitumor immunity. Sci. Immunol..

[6] Erdal E., Haider S., Rehwinkel J., Harris A.L., McHugh P.J. (2017). A prosurvival DNA damage-induced cytoplasmic interferon responseis mediated by end resection factors and is limited by Trex1. Genes Dev..

[7] Berman H.M., Westbrook J., Feng Z., Gilliland G., Bhat T.N., Weissig H., Shindyalov I.N., Bourne P.E. (2000). The Protein DataBank. Nucleic Acids Res..

[8] Isaacs A., Lindenmann J. (1957). Virus interference. I. The interferon. Proc. R. Soc. Lond. B Biol. Sci..

[9] Platanias L.C. (2005). Mechanisms of type-I- and type-II-interferon-mediated signalling. Nat. Rev. Immunol..

[10] Kotredes K.P., Gamero A.M. (2013). Interferons as inducers of apoptosis in malignant cells. J. Interferon Cytokine Res..

[11] Gresser I., Maury C., Brouty-Boyé D. (1972). Mechanism of the antitumou reffect of interferon in mice. Nature.

[12] Kaplan D.H., Shankaran V., Dighe A.S., Stockert E., Aguet M., Old L.J., Schreiber R.D. (1998). Demonstration of an interferon dependent tumor surveillance system in immunocompetent mice. Proc. Natl. Acad. Sci. USA.

[13] Borden E.C., Sen G.C., Uze G., Silverman R.H., Ransohoff R.M., Foster G.R., Stark G.R. (2007). Interferons at age 50: Past, current and future impact on biomedicine. Nat. Rev. Drug Discov..

[14] Walter M.R. (2020). The role of structure in the biology of interferon signaling. Front. Immunol..

[15] Ivashkiv L.B., Donlin L.T. (2014). Regulation of type I interferon responses. Nat. Rev. Immunol..

[16] Staeheli P. (1990). Interferon induced proteins and the antiviral state. Adv. Virus Res..

[17] Thomas C., Moraga I., Levin D., Krutzik P.O., Podoplelova Y., Trejo A., Lee C., Yarden G., Vleck S.E., Glenn J.S. (2011). Structural linkage between ligand discrimination and receptor activation by type I interferons. Cell.

[18] Mendoza J.L., Escalante N.K., Jude K.M., SotolongoBellon J., Su L., Horton T.M., Tsutsumi N., Berardinelli S.J., Haltiwanger R.S., Piehler J. (2019). Structure of the IFNγ receptor complex guides design of biased agonists. Nature.

[19] Mendoza J.L., Schneider W.M., Hoffmann H.H., Vercauteren K., Jude K.M., Xiong A., Moraga I., Horton T.M., Glenn J.S., De Jong Y.P. (2017). The IFN-λ-IFN-λR1-IL-10Rβ Complex reveals structural features underlying type III IFN functional plasticity. Immunity.

[20] Mesev E.V., LeDesma R.A., Ploss A. (2019). Decoding type I and III interferon signalling during viral infection. Nat. Microbiol..

[21] Oon S., Wilson N.J., Wicks I. (2016). Targeted therapeutics in SLE: Emerging strategies to modulate the interferon pathway. Clin. Transl. Immunol..

[22] Krause C.D., Pestka S. (2005). Evolution of the Class 2 cytokines and receptors, and discovery of new friends and relatives. Pharmacol. Ther..

[23] Novick D., Cohen B., Rubinstein M. (1994). The human interferon alpha/beta receptor: Characterization and molecular cloning. Cell.

[24] Roisman L.C., Jaitin D.A., Baker D.P., Schreiber G. (2005). Mutational analysis of the IFNAR1 binding site on IFN alpha 2 reveals the architecture of a weak ligand-receptor binding site. J. Mol. Biol..

[25] Jaitin D.A., Roisman L.C., Jaks E., Gavutis M., Piehler J., Vander Heyden J., Uze G., Schreiber G. (2006). Inquiring into the differential action of interferons (IFNs): An IFN-alpha2 mutant with enhanced affinity to IFNAR1 is functionally similar to IFN-beta. Mol. Cell Biol..

[26] Piehler J., Schreiber G. (1999). Mutational and structural analysis of the binding interface between type I interferons and their receptor Ifnar2. J. Mol. Biol..

[27] Stark G.R., Kerr I.M., Williams B.R., Silverman R.H., Schreiber R.D. (1998). How cells respond to interferons. Annu. Rev. Biochem..

[28] Hamilton J.A., Whitty G.A., Kola I., Hertzog P.J. (1996). Endogenous IFN-alpha beta suppresses colony-stimulating factor(CSF) 1 stimulated macrophage DNA synthesis and mediates inhibitory effects of lipopolysaccharide and TNF-alpha. J. Immunol..

[29] Marié I., Durbin J.E., Levy D.E. (1998). Differential viral induction of distinct interferon-alpha genes by positive feedback through interferon regulatory factor-7. EMBO J..

[30] Vogel S.N., Fertsch D. (1984). Endogenous interferon production by endotoxin-responsive macrophages provides an autostimulatory differentiation signal. Infect. Immun..

[31] Takaoka A., Mitani Y., Suemori H., Sato M., Yokochi T., Noguchi S., Tanaka N., Taniguchi T. (2000). Crosstalk between interferon-gamma and -alpha/beta signaling components in caveolar membrane domains. Science.

[32] Foster G.R., Rodrigues O., Ghouze F., Schulte-Frohlinde E., Testa D., Liao M.J., Stark G.R., Leadbeater L., Thomas H.C. (1996). Different relative activities of human cell derived interferon-alpha subtypes: IFN-alpha 8 has very high antiviral potency. J. Interferon Cytokine Res..

[33] Hibbert L., Foster G.R. (1999). Human type I interferons differ greatly in their effects on the proliferation of primary B cells. J. Interferon Cytokine Res..

[34] Sadler A.J., Williams B.R. (2008). Interferon inducible antiviral effectors. Nat. Rev. Immunol..

[35] Sistigu A., Yamazaki T., Vacchelli E., Chaba K., Enot D.P., Adam J., Vitale I., Goubar A., Baracco E.E., Remédios C. (2014). Cancer cell autonomous contribution of type I interferon signaling to the efficacy of chemotherapy. Nat. Med..

[36] Deng L., Liang H., Xu M., Yang X., Burnette B., Arina A., Li X.D., Mauceri H., Beckett M., Darga T. (2014). STING-dependent cytosolic DNA sensing promotes radiation induced Type I interferon dependent antitumor immunity in immunogenic tumors. Immunity.

[37] Yang H., Wang H., Ren J., Chen Q., Chen Z.J. (2017). cGAS is essential for cellular senescence. Proc. Natl. Acad. Sci. USA.

[38] Gasser S., Zhang W.Y.L., Tan N.Y.J., Tripathi S., Suter M.A., Chew Z.H., Khatoo M., Ngeow J., Cheung F.S.G. (2017). Sensing of dangerous DNA. Mech. Ageing Dev..

[39] Hong C., Tijhuis A.E., Foijer F. (2019). The cGAS Paradox: Contrasting roles for cGAS-STING pathway in chromosomal instability. Cells.

[40] Nastasi C., Mannarino L., D’Incalci M. (2020). DNA damage response and immune defense. Int. J. Mol. Sci..

[41] Jiang M., Chen P., Wang L., Li W., Chen B., Liu Y., Wang H., Zhao S., Ye L., He Y. (2020). cGAS-STING, an important pathway in cancer immunotherapy. J. Hematol. Oncol..

[42] Luecke S., Holleufer A., Christensen M.H., Jønsson K.L., Boni G.A., Sørensen L.K., Johannsen M., Jakobsen M.R., Hartmann R., Paludan S.R. (2017). cGAS is activated by DNA in a length-dependent manner. EMBO Rep..

[43] Marcus A., Mao A.J., Lensink-Vasan M., Wang L., Vance R.E., Raulet D.H. (2018). Tumor derived cGAMP triggers a STING mediated interferon response in non-tumor cells to activate the NK cell response. Immunity.

[44] Kanda N., Shimizu T., Tada Y., Watanabe S. (2007). IL-18 enhances IFN-γ induced production of CXCL9, CXCL10, and CXCL11 in humank eratinocytes. Eur. J. Immunol..

[45] Yu H., Pardoll D., Jove R. (2009). STATs in cancer inflammation and immunity: A leading role for STAT3. Nat. Rev. Cancer.

[46] Jones L.M., Broz M.L., Ranger J.J., Ozcelik J., Ahn R., Zuo D., Ursini-Siegel J., Hallett M.T., Krummel M., Muller W.J. (2016). STAT3 Establishes an immuno suppressive micro environment during the early stages of breast carcinogenes is to promote tumor growth and metastasis. Cancer Res..

[47] Kortylewski M., Kujawski M., Wang T., Wei S., Zhang S., Pilon-Thomas S., Niu G., Kay H., Mulé J., Kerr W.G. (2005). Inhibiting Stat3 signaling in the hematopoietic system elicits multi component antitumor immunity. Nat. Med..

[48] Grégoire C., Chasson L., Luci C., Tomasello E., Geissmann F., Vivier E., Walzer T. (2007). The trafficking of natural killer cells. Immunol. Rev..

[49] Elsea C.R., Roberts D.A., Druker B.J., Wood L.J. (2008). Inhibition of p38MAPK suppresses inflammatory cytokine induction by Etoposide, 5-Fluorouracil, and Doxorubicin without affecting tumoricidal activity. PLoS ONE.

[50] Li A., Yi M., Qin S., Song Y., Chu Q., Wu K. (2019). Activating cGAS-STING pathway for the optimal effect of cancer immunotherapy. J. Hematol. Oncol..

[51] Parkes E.E., Walker S.M., Taggart L.E., McCabe N., Knight L.A., Wilkinson R., McCloskey K.D., Buckley N.E., Savage K.I., Salto-Tellez M. (2017). Activation of STING-dependent innate immune signaling by S-Phase specific DNA damage in breast cancer. J. Natl. Cancer Inst..

[52] Harding S.M., Benci J.L., Irianto J., Discher D.E., Minn A.J., Greenberg R.A. (2017). Mitotic progression following DNA damage enables pattern recognition within micro nuclei. Nature.

[53] Gerber S.A., Sedlacek A.L., Cron K.R., Murphy S.P., Frelinger J.G., Lord E.M. (2013). IFN-γ mediates the antitumor effects of radiation therapy in a murine colon tumor. Am. J. Pathol..

[54] Tanimoto T., Yamamoto S., Taniai M., Taniguchi M., Ariyasu H., Ushio C., Aga M., Mukai Y., Tsutsumi Y., Ariyasu T. (2007). The combination of IFN-alpha 2 and IFN-alpha 8 exhibits synergistic antiproliferative activity on renal cell carcinoma (RCC) cell lines through increased binding affinity for IFNAR-2. J. Interferon Cytokine Res..

[55] DiFranco S., Turdo A., Todaro M., Stassi G. (2017). Role of Type I and II Interferons in colorectal cancer and melanoma. Front. Immunol..

[56] Minn A.J. (2015). Interferons and the immunogenic effects of cancer therapy. Trends Immunol..

[57] Khodarev N.N., Beckett M., Labay E., Darga T., Roizman B., Weichselbaum R.R. (2004). STAT1 is overexpressed in tumors selected for radioresistance and confers protection from radiation in transduced sensitive cells. Proc. Natl. Acad. Sci. USA.

[58] Harris A.L. (2002). Hypoxia- a key regulatory factor in tumour growth. Nat. Rev. Cancer.

[59] Hallahan D.E., Haimovitz-Friedman A., Kufe D.W., Fuks Z., Weichselbaum R.R. (1993). The role of cytokines in radiation oncology. Important Adv. Oncol..

[60] Marples B., Scott S.D., Hendry J.H., Embleton M.J., Lashford L.S., Margison G.P. (2000). Development of synthetic promoters for radiation-mediated gene therapy. Gene Ther..

[61] Kita K., Sugaya S., Zhai L., Wu Y.P., Wano C., Chigira S., Nomura J., Takahashi S., Ichinose M., Suzuki N. (2003). Involvement of LEU13 in interferon-induced refractoriness of human RSa cells to cell killing by Xrays. Radiat. Res..

[62] Benci J.L., Johnson L.R., Choa R., Xu Y., Qiu J., Zhou Z., Xu B., Ye D., Nathanson K.L., June C.H. (2019). Opposing functions of interferon coordinate adaptive and innate immune responses to cancer immune check point blockade. Cell.

[63] Duarte C.W., Willey C.D., Zhi D., Cui X., Harris J.J., Vaughan L.K., Mehta T., McCubrey R.O., Khodarev N.N., Weichselbaum R.R. (2012). Expression signature of IFN/STAT1 signaling genes predicts poor survival outcome in glioblastoma multiforme in a subtype-specific manner. PLoS ONE.

[64] Tsai M.H., Cook J.A., Chandramouli G.V., DeGraff W., Yan H., Zhao S., Coleman C.N., Mitchell J.B., Chuang E.Y. (2007). Gene expression profiling of breast, prostate, and glioma cells following single versus fractionated doses of radiation. Cancer Res..

[65] Liauw S.L., Connell P.P., Weichselbaum R.R. (2013). New paradigms and future challenges in radiation oncology: An update of biological targets and technology. Sci. Transl. Med..

[66] Khodarev N.N., Minn A.J., Efimova E.V., Darga T.E., Labay E., Beckett M., Mauceri H.J., Roizman B., Weichselbaum R.R. (2007). Signal transducer and activator of transcription 1 regulates both cytotoxic and prosurvival functions in tumor cells. Cancer Res..

[67] Cheon H., Stark G.R. (2009). Unphosphorylated STAT1 prolongs the expression of interferon-induced immune regulatory genes. Proc. Natl. Acad. Sci. USA.

[68] Cheon H., Yang J., Stark G.R. (2011). The functions of signal transducers and activators of transcriptions 1 and 3 as cytokine-inducible proteins. J. Interferon Cytokine Res..

[69] Cheon H., Holvey-Bates E.G., Schoggins J.W., Forster S., Hertzog P., Imanaka N., Rice C.M., Jackson M.W., Junk D.J., Stark G.R. (2013). IFNβ dependent increases in STAT1, STAT2, and IRF9 mediate resistance to viruses and DNA damage. EMBO J..

[70] Khodarev N.N., Roach P., Pitroda S.P., Golden D.W., Bhayani M., Shao M.Y., Darga T.E., Beveridge M.G., Sood R.F., Sutton H.G. (2009). STAT1 pathway mediates amplification of metastatic potential and resistance to therapy. PLoS ONE.

[71] Khodarev N.N., Roizman B., Weichselbaum R.R. (2012). Molecular pathways: Interferon/stat1 pathway: Role in the tumor resistance to genotoxic stress and aggressive growth. Clin. Cancer Res..

[72] Takeuchi O., Akira S. (2010). Pattern recognition receptors and inflammation. Cell.

[73] Mackenzie K.J., Carroll P., Martin C.A., Murina O., Fluteau A., Simpson D.J., Olova N., Sutcliffe H., Rainger J.K., Leitch A. (2017). cGAS surveillance of micronuclei links genome instability to innate immunity. Nature.

[74] Cheon H., Borden E.C., Stark G.R. (2014). Interferons and their stimulated genes in the tumor microenvironment. Semin. Oncol..

[75] Weichselbaum R.R., Ishwaran H., Yoon T., Nuyten D.S., Baker S.W., Khodarev N., Su A.W., Shaikh A.Y., Roach P., Kreike B. (2008). An interferon-related gene signature for DNA damage resistance is a predictive marker for chemotherapy and radiation for breast cancer. Proc. Natl. Acad. Sci. USA.

[76] Wallace T.A., Martin D.N., Ambs S. (2011). Interactions among genes, tumor biology and the environment in cancer health disparities: Examining the evidence on a national and global scale. Carcinogenesis.

[77] UniProt Consortium (2019). UniProt: A worldwide hub of protein knowledge. Nucleic Acids Res..

[78] Raudvere U., Kolberg L., Kuzmin I., Arak T., Adler P., Peterson H., Vilo J. (2019). g:Profiler: A web server for functional enrichment analysis and conversions of gene lists (2019 update). Nucleic Acids Res..

[79] Reimand J., Isserlin R., Voisin V., Kucera M., Tannus-Lopes C., Rostamianfar A., Wadi L., Meyer M., Wong J., Xu C. (2019). Pathway enrichment analysis and visualization of omics data using g:Profiler, GSEA, Cytoscape and Enrichment Map. Nat. Protoc..

[80] Shannon P., Markiel A., Ozier O., Baliga N.S., Wang J.T., Ramage D., Amin N., Schwikowski B., Ideker T. (2003). Cytoscape: A software environment for integrated models of biomolecular interaction networks. Genome Res..

[81] Rusinova I., Forster S., Yu S., Kannan A., Masse M., Cumming H., Chapman R., Hertzog P.J. (2013). Interferome v2.0: An updated database of annotated interferon-regulated genes. Nucleic Acids Res..

[82] Stark G.R., Darnell J.E. (2012). The JAK-STAT pathway at twenty. Immunity.

[83] Savoldi-Barbosa M., Sakamoto-Hojo E.T. (2001). Influence of interferon-gamma on radiation-induced apoptosis in normal and a taxia-telangiectasia fibroblast cell lines. Teratog. Carcinog. Mutagen..

[84] Sirota N.P., Bezlepkin V.G., Kuznetsova E.A., Lomayeva M.G., Milonova I.N., Ravin V.K., Gaziev A.I., Bradbury R.J. (1996). Modifying effect in vivo of interferon alpha on induction and repair of lesions of DNA of lymphoid cells of gamma-irradiated mice. Radiat. Res..

[85] Wu B., Hur S. (2015). How RIG-I like receptors activate MAVS. Curr. Opin. Virol..

[86] Boelens M.C., Wu T.J., Nabet B.Y., Xu B., Qiu Y., Yoon T., Azzam D.J., Twyman-SaintVictor C., Wiemann B.Z., Ishwaran H. (2014). Exosome transfer from stromal to breast cancer cells regulates therapy resistance pathways. Cell.

[87] Gonzalez K.J., Moncada-Giraldo D.M., Gutierrez J.B. (2020). In silico identification of potential inhibitors against human 2′-5′-oligoadenylate synthetase (OAS) proteins. Comput. Biol. Chem..

[88] Souissi I., Ladam P., Cognet J.A., LeCoquil S., Varin-Blank N., Baran-Marszak F., Metelev V., Fagard R. (2012). A STAT3-inhibitory hairpin decoy oligodeoxy nucleotide discriminates between STAT1 and STAT3 and induces death in a human colon carcinoma cell line. Mol. Cancer.

[89] Joo C.H., Shin Y.C., Gack M., Wu L., Levy D., Jung J.U. (2007). Inhibition of interferon regulatory factor7 (IRF7) mediated interferon signal transduction by the Kaposi’s sarcoma-associated herpes virus viral IRF homolog vIRF3. J. Virol..

[90] Kalathiya U., Padariya M., Faktor J., Coyaud E., Alfaro J.A., Fahraeus R., Hupp T.R., Goodlett D.R. (2021). Interfaces with structure dynamics of the workhorses from cells revealed through cross-linking mass spectrometry (CLMS). Biomolecules.

[91] Chen X., Vinkemeier U., Zhao Y., Jeruzalmi D., Darnell J.E., Kuriyan J. (1998). Crystal structure of atyrosine phosphorylated STAT-1 dimer bound to DNA. Cell.

[92] Ibsen M.S., Gad H.H., Andersen L.L., Hornung V., Julkunen I., Sarkar S.N., Hartmann R. (2015). Structural and functional analysis reveals that human OASL binds dsRNA to enhance RIG-I signaling. Nucleic Acids Res..

[93] Panne D., Maniatis T., Harrison S.C. (2007). An atomic model of the interferon-beta enhanceosome. Cell.

[94] Dar A.C., Dever T.E., Sicheri F. (2005). Higher-order substrate recognition of eIF2 alpha by the RNA-dependent protein kinase PKR. Cell.

[95] Chen M.H., Ben-Efraim I., Mitrousis G., Walker-Kopp N., Sims P.J., Cingolani G. (2005). Phospholipid scramblase 1 contains a nonclassical nuclear localization signal with unique binding site in importin alpha. J. Biol. Chem..

[96] Wu B., Peisley A., Richards C., Yao H., Zeng X., Lin C., Chu F., Walz T., Hur S. (2013). Structural basis for dsRNA recognition, filament formation, and antiviral signal activation by MDA5. Cell.

[97] Yang H., Wang J., Jia X., McNatt M.W., Zang T., Pan B., Meng W., Wang H.W., Bieniasz P.D., Xiong Y. (2010). Structural insight into the mechanisms of enveloped virus tethering by tetherin. Proc. Natl. Acad. Sci. USA.

[98] Abbas Y.M., Laudenbach B.T., Martínez-Montero S., Cencic R., Habjan M., Pichlmair A., Damha M.J., Pelletier J., Nagar B. (2017). Structure of human IFIT1 with capped RNA reveals adaptable mRNA binding and mechanisms for sensing N1 and N2 ribose 2′-Omethylations. Proc. Natl. Acad. Sci. USA.

[99] Donovan J., Whitney G., Rath S., Korennykh A. (2015). Structural mechanism of sensing long dsRNA via an oncatalytic domain in human oligoadenylate synthetase 3. Proc. Natl. Acad. Sci. USA.

[100] Donovan J., Dufner M., Korennykh A. (2013). Structural basis for cytosolic double-stranded RNA surveillance by human oligoadenylate synthetase 1. Proc. Natl. Acad. Sci. USA.

[101] Rennie M.L., McKelvie S.A., Bulloch E.M., Kingston R.L. (2014). Transient dimerization of human MxA promotes GTP hydrolysis, resulting in a mechanical power stroke. Structure.

[102] Fribourgh J.L., Nguyen H.C., Matreyek K.A., Alvarez F.J.D., Summers B.J., Dewdney T.G., Aiken C., Zhang P., Engelman A., Xiong Y. (2014). Structural insight into HIV-1 restriction by MxB. Cell Host Microbe.

[103] Mazza C. (1997). Human Type I 17 Beta-Hydroxysteroid Dehydrogenase: Site Directed Mutagenesis and X-ray Crystallography Structure Function Analysis. Ph.D. Thesis.

[104] Wathelet M.G., Lin C.H., Parekh B.S., Ronco L.V., Howley P.M., Maniatis T. (1998). Virus infection induces the assembly of coordinately activated transcription factors on the IFN-beta enhancer in vivo. Mol. Cell.

[105] Yang H., Lin C.H., Ma G., Baffi M.O., Wathelet M.G. (2003). Interferon regulatory factor-7 synergizes with other transcription factors through multiple interactions with p300/CBP coactivators. J. Biol. Chem..

[106] Ning S., Pagano J.S., Barber G.N. (2011). IRF7: Activation, regulation, modification and function. Genes Immun..

[107] Lin R., Génin P., Mamane Y., Hiscott J. (2000). Selective DNA binding and association with the CREB binding protein coactivator contribute to differential activation of alpha/beta interferon genes by interferon regulatory factors 3 and 7. Mol. Cell Biol..

[108] Kileng O., Bergan V., Workenhe S.T., Robertsen B. (2009). Structural and functional studies of an IRF-7-like gene from Atlantics almon. Dev. Comp. Immunol..

[109] Kristiansen H., Gad H.H., Eskildsen-Larsen S., Despres P., Hartmann R. (2011). The oligoadenylate synthetase family: An ancient protein family with multiple antiviral activities. J. Interferon Cytokine Res..

[110] Kristiansen H., Scherer C.A., McVean M., Iadonato S.P., Vends S., Thavachelvam K., Steffensen T.B., Horan K.A., Kuri T., Weber F. (2010). Extracellular 2′-5′ oligoadenylate synthetase stimulates RNaseL-independent antiviral activity: A novel mechanism of virus induced innate immunity. J. Virol..

[111] Latham K.E., Cosenza S., Reichenbach N.L., Mordechai E., Adelson M.E., Kon N., Horvath S.E., Charubala R., Mikhailov S.N., Pfeiderer W. (1996). Inhibition of growth of estrogen receptor positive and estrogen receptor negative breast cancer cells in culture by AA-etherA, a stable 2-5A derivative. Oncogene.

[112] Feng Z., Zheng W., Tang Q., Cheng L., Li H., Ni W., Pan X. (2017). Fludarabine inhibits STAT1-mediated up-regulation of caspase-3 expression indexamethasone-induced osteoblasts apoptosis and slows the progression of steroid induced a vascular necrosis of the femoral head in rats. Apoptosis.

[113] Gunning P.T., Katt W.P., Glenn M., Siddiquee K., Kim J.S., Jove R., Sebti S.M., Turkson J., Hamilton A.D. (2007). Isoform selective inhibition of STAT1 or STAT3 homo-dimerization via peptidomimetic probes: Structural recognition of STAT SH2 domains. Bioorg. Med. Chem. Lett..

[114] Zhou X.X., Gao P.J., Sun B.G. (2009). Pravastatin attenuates interferon-gamma action via modulation of STAT1 to prevent aortic atherosclerosis in apolipoprotein E-knockout mice. Clin. Exp. Pharmacol. Physiol..

[115] Böhmer F.D., Friedrich K. (2014). Protein tyrosine phosphatases as wardens of STAT signaling. JAKSTAT.

[116] Porritt R.A., Hertzog P.J. (2015). Dynamic control of type I IFN signalling by an integrated network of negative regulators. Trends Immunol..

[117] Rytinki M.M., Kaikkonen S., Pehkonen P., Jääskeläinen T., Palvimo J.J. (2009). PIA Sproteins: Pleiotropic interactors associated with SUMO. Cell. Mol. Lif eSci..

[118] Usmani S.Z., Sexton R., Ailawadhi S., Shah J.J., Valent J., Rosenzweig M., Lipe B., Zonder J.A., Fredette S., Durie B. (2015). Phase I safety data of lenalidomide, bortezomib, dexamethasone, and elotuzumab as induction therapy for newly diagnosed symptomatic multiple myeloma: SWOG S1211. Blood Cancer J..

[119] Lan Q., Peyvandi S., Duffey N., Huang Y.T., Barras D., Held W., Richard F., Delorenzi M., Sotiriou C., Desmedt C. (2019). Type I interferon/IRF7 axis instigates chemotherapy-induced immunological dormancy in breastc ancer. Oncogene.

[120] Liang Q., Fu B., Wu F., Li X., Yuan Y., Zhu F. (2012). ORF45 of Kaposi’s sarcoma-associated herpes virus inhibits phosphorylation of interferon regulatory factor 7 by IKKε and TBK1 as an alternatives ubstrate. J. Virol..

[121] Jammi N.V., Whitby L.R., Beal P.A. (2003). Small molecule inhibitors of the RNA-dependent protein kinase. Biochem. Biophys. Res. Commun..

[122] Sanfilippo C., Pinzone M.R., Cambria D., Longo A., Palumbo M., DiMarco R., Condorelli F., Nunnari G., Malaguarnera L., DiRosa M. (2018). OAS gene family expression is associated with HIV-Related neurocognitive disorders. Mol. Neurobiol..

[123] Field L.L., Bonnevie-Nielsen V., Pociot F., Lu S., Nielsen T.B., Beck-Nielsen H. (2005). OAS1 splice site polymorphism controlling antiviral enzyme activity influences susceptibility to type 1 diabetes. Diabetes.

[124] Banerjee S., Gusho E., Gaughan C., Dong B., Gu X., Holvey-Bates E., Talukdar M., Li Y., Weiss S.R., Sicheri F. (2019). OAS-RNase L innate immune pathway mediates the cytotoxicity of a DNA-demethylating drug. Proc. Natl. Acad. Sci. USA.

[125] Kodigepalli K.M., Bowers K., Sharp A., Nanjundan M. (2015). Roles and regulation of phospholipid scramblases. FEBS Lett..

[126] Tufail Y., Cook D., Fourgeaudm L., Powers C.J., Merten K., Clark C.L., Hoffman E., Ngo A., Sekiguchi K.J., O’Shea C.C. (2017). Phosphatidyl serine exposure controls viral innate immune responses by microglia. Neuron.

[127] Chow K.T., Gale M., Loo Y.M. (2018). RIG-I and other RNA sensors in antiviral immunity. Annu. Rev. Immunol..

[128] Kasumba D.M., Hajake T., Oh S.W., Kotenko S.V., Kato H., Fujita T. (2017). A Plant-derived nucleic acid reconciles type I IFN and a Pyroptotic-like event in immunity against respiratory viruses. J. Immunol..

[129] Oberson A., Spagnuolo L., Puddinu V., Barchet W., Rittner K., Bourquin C. (2017). NAB2 is a novel immune stimulator of MDA-5 that promotes a strong type I interferon response. Oncotarget.

[130] Johnson B., VanBlargan L.A., Xu W., White J.P., Shan C., Shi P.Y., Zhang R., Adhikari J., Gross M.L., Leung D.W. (2018). Human IFIT3 modulates IFIT1 RNA binding specificity and protein stability. Immunity.

[131] Pidugu V.K., Wu M.M., Yen A.H., Pidugu H.B., Chang K.W., Liu C.J., Lee T.C. (2019). IFIT1 and IFIT3 promote oral squamous cell carcinoma metastasis and contribute to the anti-tumor effect of gefitinib via enhancing p-EGFR recycling. Oncogene.

[132] Mahauad-Fernandez W.D., Okeoma C.M. (2018). B49, a BST-2-based peptide, inhibits adhesion and growth of breast cancer cells. Sci. Rep..

[133] Cheng Y., Ma X.L., Wei Y.Q., Wei X.W. (2019). Potential roles and targeted therapy of the CXCLs/CXCR2 axis in cancer and inflammatory diseases. Biochim. Biophys. Acta Rev. Cancer.

[134] Chrétien I., Marcuz A., Courtet M., Katevuo K., Vainio O., Heath J.K., White S.J., DuPasquier L. (1998). CTX, a Xenopus thymocyte receptor, defines a molecular family conserved throughout vertebrates. Eur. J. Immunol..

[135] Padariya M., Kalathiya U., Mikac S., Dziubek K., Tovar Fernandez M.C., Sroka E., Fahraeus R., Sznarkowska A. (2021). Viruses, cancer and non-self recognition. Open Biol..

[136] Villarreal L.P. (2009). The source of self: Genetic parasites and the origin of adaptive immunity. Ann. N. Y. Acad. Sci..

[137] Chuong E.B., Elde N.C., Feschotte C. (2016). Regulatory evolution of innate immunity through co-option of endogenous retroviruses. Science.

[138] Didcock L., Young D.F., Goodbourn S., Randall R.E. (1999). The V protein of simianvirus 5 inhibits interferon signalling by targeting STAT1 for proteasome-mediated degradation. J. Virol..

[139] Andrejeva J., Young D.F., Goodbourn S., Randall R.E. (2002). Degradation of STAT1 and STAT2 by the V proteins of simianvirus 5 and human parainfluenza virus type 2, respectively: Consequences for virus replication in the presence of alpha/beta and gamma interferons. J. Virol..

[140] Look D.C., Roswit W.T., Frick A.G., Gris-Alevy Y., Dickhaus D.M., Walter M.J., Holtzman M.J. (1998). Direct suppression of Stat1 function during adenoviral infection. Immunity.

[141] Najarro P., Traktman P., Lewis J.A. (2001). Vaccinia virus blocks gamma interferon signal transduction: Viral VH1 phosphatase reverses Stat1 activation. J. Virol..

[142] Arbiza J., Mirazo S., Fort H. (2010). Viral quasispecies profiles as the result of the interplay of competition and cooperation. BMC Evol. Biol..

[143] Ojosnegros S., Perales C., Mas A., Domingo E. (2011). Quasispecies as a matter of fact: Viruses and beyond. Virus Res..

[144] Lauring A.S., Andino R. (2010). Quasispecies theory and the behavior of RNA viruses. PLoS Pathog..

[145] Villarreal L.P., Witzany G. (2013). Rethinking quasispecies theory: From fittest type to cooperative consortia. World J. Biol. Chem..

[146] Domingo E., Perales C. (2019). Viral quasispecies. PLoS Genet..

[147] Huang W.T., Lin C.W. (2014). EBV-encoded miR-BART20-5p and miR-BART8 inhibitthe IFN-γ-STAT1 pathway associated with disease progression in nasal NK-cell lymphoma. Am. J. Pathol..

[148] Drappier M., Michiels T. (2015). Inhibition of the OAS/RNaseL pathway by viruses. Curr. Opin. Virol..

[149] Han J.Q., Townsend H.L., Jha B.K., Paranjape J.M., Silverman R.H., Barton D.J. (2007). A phylogenetically conserved RNA structure in the poliovirus open reading frame inhibits the antiviral endoribonuclease RNaseL. J. Virol..

[150] Han J.Q., Barton D.J. (2002). Activation and evasion of the antiviral 2′-5′ oligoadenylate synthetase/ribonucleaseL pathway by hepatitis C virus mRNA. RNA.

[151] Min J.Y., Krug R.M. (2006). The primary function of RNA binding by the influenza a virus NS1 protein in infected cells: Inhibiting the 2′-5′ oligo(A)synthetase/RNaseL pathway. Proc. Natl. Acad. Sci. USA.

[152] Chang H.W., Watson J.C., Jacobs B.L. (1992). The E3L gene of vaccinia virus encodes an inhibitor of the interferon-induced, double-stranded RNA-dependent protein kinase. Proc. Natl. Acad. Sci. USA.

[153] Huismans H., Joklik W.K. (1976). Reovirus-coded polypeptides in infected cells: Isolation of two native monomeric polypeptides with affinity for single-stranded and double-stranded RNA, respectively. Virology.

[154] Schröder H.C., Ugarković D., Wenger R., Reuter P., Okamoto T., Müller W.E. (1990). Binding of Tat protein to TAR region of human immunodeficiency virus type 1 blocks TAR-mediated activation of (2′-5′)oligoadenylate synthetase. AIDS Res. Hum. Retrovir..

[155] Burgess H.M., Mohr I. (2015). Cellular 5′-3′ mRNA exonuclease Xrn1 controls double-stranded RNA accumulation and anti-viral responses. Cell Host Microbe.

[156] Liu S.W., Katsafanas G.C., Liu R., Wyatt L.S., Moss B. (2015). Poxvirus decapping enzymes enhance virulence by preventing the accumulation of dsRNA and the induction of innate antiviral responses. Cell Host Microbe.

[157] Zhao L., Jha B.K., Wu A., Elliott R., Ziebuhr J., Gorbalenya A.E., Silverman R.H., Weiss S.R. (2012). Antagonism of the interferon-induced OAS-RNaseL pathway by murine coronavirus ns2 protein is required for virus replication and liver pathology. Cell Host Microbe.

[158] Zhang R., Jha B.K., Ogden K.M., Dong B., Zhao L., Elliott R., Patton J.T., Silverman R.H., Weiss S.R. (2013). Homologous 2′,5′-phosphodiesterases from disparate RNA viruses antagonize antiviral innate immunity. Proc. Natl. Acad. Sci. USA.

[159] Ogden K.M., Hu L., Jha B.K., Sankaran B., Weiss S.R., Silverman R.H., Patton J.T., Prasad B.V. (2015). Structural basis for 2′-5′-oligoadenylate binding and enzyme activity of aviral RNaseL antagonist. J. Virol..

[160] Cayley P.J., Davies J.A., McCullagh K.G., Kerr I.M. (1984). Activation of the ppp(A2′p)nA system in interferon-treated, herpes simplex virus-infected cells and evidence for novel inhibitors of the ppp(A2′p)nA-dependent RNase. Eur. J. Biochem..

[161] Kato H., Takeuchi O., Sato S., Yoneyama M., Yamamoto M., Matsui K., Uematsu S., Jung A., Kawai T., Ishii K.J. (2006). Differential roles of MDA5 and RIG-I helicases in the recognition of RNA viruses. Nature.

[162] Hornung V., Ellegast J., Kim S., Brzózka K., Jung A., Kato H., Poeck H., Akira S., Conzelmann K.K., Schlee M. (2006). 5′-Triphosphate RNA is the ligand for RIG-I. Science.

[163] Peisley A., Lin C., Wu B., Orme-Johnson M., Liu M., Walz T., Hur S. (2011). Cooperative assembly and dynamic disassembly of MDA5 filaments for viral dsRNA recognition. Proc. Natl. Acad. Sci. USA.

[164] Gorman J.A., Hundhausen C., Errett J.S., Stone A.E., Allenspach E.J., Ge Y., Arkatkar T., Clough C., Dai X., Khim S. (2017). The A946T variant of the RNA sensor IFIH1 mediates an interferon program that limits viral infection but increases the risk for autoimmunity. Nat. Immunol..

[165] Leung D.W., Shabman R.S., Farahbakhsh M., Prins K.C., Borek D.M., Wang T., Mühlberger E., Basler C.F., Amarasinghe G.K. (2010). Structural and functional characterization of Reston Ebola virus VP35 interferon inhibitory domain. J. Mol. Biol..

[166] Ramanan P., Edwards M.R., Shabman R.S., Leung D.W., Endlich-Frazier A.C., Borek D.M., Otwinowski Z., Liu G., Huh J., Basler C.F. (2012). Structural basis for Marburg virus VP35-mediated immune evasion mechanisms. Proc. Natl. Acad. Sci. USA.

[167] Motz C., Schuhmann K.M., Kirchhofer A., Moldt M., Witte G., Conzelmann K.K., Hopfner K.P. (2013). Paramyxovirus V proteins disrupt the fold of the RNA sensor MDA5 to inhibit antiviral signaling. Science.

[168] Davis M.E., Wang M.K., Rennick L.J., Full F., Gableske S., Mesman A.W., Gringhuis S.I., Geijtenbeek T.B., Duprex W.P., Gack M.U. (2014). Antagonism of the phosphatase PP1 by the measles virus V protein is required for innate immune escape of MDA5. Cell Host Microbe.

[169] Chatterjee S., Basler C.F., Amarasinghe G.K., Leung D.W. (2016). Molecular Mechanisms of innate immune inhibition bynon-segmented negative-sense RNA viruses. J. Mol. Biol..

[170] Zhuang T., Yi X., Chen J., Kang P., Chen X., Chen J., Cui T., Chang Y., Ye Z., Ni Q. (2020). Intracellular virus sensor MDA5 exacerbates vitiligo by inducing the secretion of chemokines in keratinocytes under virus invasion. Cell Death Dis..

[171] Bailey C.C., Zhong G., Huang I.C., Farzan M. (2014). IFITM-family Proteins: The cell’s first line of antiviral defense. Annu. Rev. Virol..

[172] Brass A.L., Huang I.C., Benita Y., John S.P., Krishnan M.N., Feeley E.M., Ryan B.J., Weyer J.L., Vander Weyden L., Fikrig E. (2009). The IFITM proteins mediate cellular resistance to influenza AH1N1 virus, West Nilevirus, and denguevirus. Cell.

[173] Huang I.C., Bailey C.C., Weyer J.L., Radoshitzky S.R., Becker M.M., Chiang J.J., Brass A.L., Ahmed A.A., Chi X., Dong L. (2011). Distinct patterns of IFITM-mediated restriction of filoviruses, SARS coronavirus, and influenza A virus. PLoS Pathog..

[174] Wilkins C., Woodward J., Lau D.T., Barnes A., Joyce M., McFarlane N., McKeating J.A., Tyrrell D.L., Gale M. (2013). IFITM1 is a tight junction protein that inhibit shepatitis C virus entry. Hepatology.

[175] Habjan M., Hubel P., Lacerda L., Benda C., Holze C., Eberl C.H., Mann A., Kindler E., Gil-Cruz C., Ziebuhr J. (2013). Sequestration by IFIT1 impairs translation of 2′O-unmethylated capped RNA. PLoS Pathog..

[176] Hyde J.L., Gardner C.L., Kimura T., White J.P., Liu G., Trobaugh D.W., Huang C., Tonelli M., Paessler S., Takeda K. (2014). A viral RNA structural element alters host recognition of nonself RNA. Science.

[177] Kumar P., Sweeney T.R., Skabkin M.A., Skabkina O.V., Hellen C.U., Pestova T.V. (2014). Inhibition of translation by IFIT family members is determined by their ability to interact selectively with the 5′-terminal regions of cap0-, cap1- and 5′ppp-mRNAs. Nucleic Acids Res..

[178] Diamond M.S. (2014). IFIT1: A dual sens or and effect or molecule that detects non-2′-Omethylated viral RNA and inhibits its translation. Cytokine Growth Factor Rev..

[179] Wang C., Pflugheber J., Sumpter R., Sodora D.L., Hui D., Sen G.C., Gale M. (2003). Alpha interferon induces distinct translational control programs to suppress hepatitis C virus RNA replication. J. Virol..

[180] Pichlmair A., Lassnig C., Eberle C.A., Górna M.W., Baumann C.L., Burkard T.R., Bürckstümmer T., Stefanovic A., Krieger S., Bennett K.L. (2011). IFIT1 is an antiviral protein that recognizes 5′-triphosphate RNA. Nat. Immunol..

[181] Bouloy M., Plotch S.J., Krug R.M. (1978). Globin mRNAs are primers for the transcription of influenza viral RNA in vitro. Proc. Natl. Acad. Sci. USA.

[182] Plotch S.J., Bouloy M., Ulmanen I., Krug R.M. (1981). A unique cap(m7GpppXm)-dependent influenza virion endonuclease cleaves capped RNAs to generate the primers that initiate viral RNA transcription. Cell.

[183] Pyper J.M., Clements J.E., Zink M.C. (1998). The nucleolusis the site of Borna disease virus RNA transcription and replication. J. Virol..

[184] Reynaud J.M., Kim D.Y., Atasheva S., Rasalouskaya A., White J.P., Diamond M.S., Weaver S.C., Frolova E.I., Frolov I. (2015). IFIT1 Differentially Interferes with translation and replication of alpha virus genomes and promotes induction of Type I interferon. PLoS Pathog..

[185] Li S., Labrecque S., Gauzzi M.C., Cuddihy A.R., Wong A.H., Pellegrini S., Matlashewski G.J., Koromilas A.E. (1999). The human papillomavirus(HPV)-18E6 oncoprotein physically associates with Tyk2 and impairs Jak-STAT activation by interferon-alpha. Oncogene.

[186] Zhou C., Tuong Z.K., Frazer I.H. (2019). Papillomavirus immune evasion strategies target the infected cell and the local immune system. Front. Oncol..

[187] Chang Y.E., Laimins L.A. (2000). Microarray analysis identifies interferon-inducible genes and Stat-1 as major transcriptional targets of human papillomavirus type 31. J. Virol..

[188] Hong S., Mehta K.P., Laimins L.A. (2011). Suppression of STAT-1 expression by human papillomaviruses is necessary for differentiation-dependent genome amplification and plasmid maintenance. J. Virol..

[189] Nees M., Geoghegan J.M., Hyman T., Frank S., Miller L., Woodworth C.D. (2001). Papillomavirus type 16 oncogenes downregulate expression of interferon-responsive genes and upregulate proliferation-associated and NF-kappaB-responsive genes in cervical keratinocytes. J. Virol..

[190] Barnard P., McMillan N.A. (1999). The human papillomavirus E7 oncoprotein abrogates signaling mediated by interferon-alpha. Virology.

[191] Barnard P., Payne E., McMillan N.A. (2000). The human papillomavirus E7 protein is able to inhibit the antiviral and anti-growth functions of interferon-alpha. Virology.

[192] Boccardo E., Lepique A.P., Villa L.L. (2010). The role of inflammation in HPV carcinogenesis. Carcinogenesis.

[193] Schneider A., Papendick U., Gissmann L., DeVilliers E.M. (1987). Interferon treatment of human genital papillomavirus infection: Importance of viral type. Int. J. Cancer.

[194] Rincon-Orozco B., Halec G., Rosenberger S., Muschik D., Nindl I., Bachmann A., Ritter T.M., Dondog B., Ly R., Bosch F.X. (2009). Epigenetic silencing of interferon-kappa in human papillomavirus type 16-positive cells. Cancer Res..

